# Evolutionary patterns of chimeric retrogenes in *Oryza* species

**DOI:** 10.1038/s41598-019-54085-2

**Published:** 2019-11-27

**Authors:** Yanli Zhou, Chengjun Zhang

**Affiliations:** 1 The Germplasm Bank of Wild Species, Kunming Institute of Botany, Chinese Academy of Sciences, No. 132 Lanhei Road, Kunming, 650201 Yunnan China; 2 Haiyan Engineering & Technology Center, Kunming Institute of Botany, Chinese Academy of Science, Jiaxing, 314300 Zhejiang China

**Keywords:** Molecular evolution, Plant evolution

## Abstract

Chimeric retroposition is a process by which RNA is reverse transcribed and the resulting cDNA is integrated into the genome along with flanking sequences. This process plays essential roles and drives genome evolution. Although the origination rates of chimeric retrogenes are high in plant genomes, the evolutionary patterns of the retrogenes and their parental genes are relatively uncharacterised in the rice genome. In this study, we evaluated the substitution ratio of 24 retrogenes and their parental genes to clarify their evolutionary patterns. The results indicated that seven gene pairs were under positive selection. Additionally, soon after new chimeric retrogenes were formed, they rapidly evolved. However, an unexpected pattern was also revealed. Specifically, after an undefined period following the formation of new chimeric retrogenes, the parental genes, rather than the new chimeric retrogenes, rapidly evolved under positive selection. We also observed that one retro chimeric gene (*RCG3*) was highly expressed in infected calli, whereas its parental gene was not. Finally, a comparison of our Ka/Ks analysis with that of other species indicated that the proportion of genes under positive selection is greater for chimeric retrogenes than for non-chimeric retrogenes in the rice genome.

## Introduction

Retroposed gene copies (i.e., retrogenes) are the result of a retrotransposition, which refers to a process in which mRNAs sequences are reverse-transcribed into cDNA, which is then inserted into a new genomic position^1^. Because of the processed nature of mRNAs, the newly duplicated paralogs lack introns and contain a poly-A tail as well as short flanking repeats, leading to the functional inefficiency of retrogenes due to a lack of regulatory elements. However, retro chimeric genes (RCG) are associated with increased gene integrity via the recruitment of genomic flanking sequence, enabling the development of new functions that contribute to adaptive evolution.

The gene *Jingwei*, which originated by the insertion of a retrocopy of the Alcohol dehydrogenase gene (*Adh*) into the *yande* in *Drosophila* species, represents the first characterized young chimeric gene^2^. Many other retrogenes with chimeric structures have since been reported in animals, including the *Sdic* gene that resulted from the fusion between *Cdic* and *AnnX*^3^, the non-protein-coding RNA gene *sphinx*^4^, the retroposed fission gene family *monkey king* (*mkg*)^5^, and the *siren* gene derived from *Adh*^6^. Additionally, 14 chimeric genes were identified in *Drosophila* species^7^, including one (*Qtzl*) affecting the male reproductive system^8^. Moreover, approximately 20 retrogenes have been identified in primates and other mammals^9^. For example, the gene encoding the TRIM5-CypA fusion protein (*TRIMCyp*) formed because of a transposition of the cyclophilin A cDNA (*CypA*) into the *TRIM5* locus^10,11^. Marques determined that approximately 57 retrogenes detected in the human genome originated in primates^12^. In contrast to the considerable research that has been conducted on animal retrogenes, plant retrogenes were not systematically identified until the *Arabidopsis thaliana* retroposons were identified^1^. Chimeric retrogenes were subsequently detected in rice^13^. In the rice genome, abundant retroposition-mediated chromosomal rearrangements resulted in 898 putative retrogenes, 380 of which generated chimeric gene structures following the recruiting nearby exon-intron sequences. Many of these chimeric retrogenes originated recently, while their evolutionary trajectories remain poorly understood.

Technical advances have made it easier to search for new retrogenes, and provided opportunities for researchers to thoroughly investigate the evolutionary patterns of chimeric retrogenes. There are reports describing parallel changes in the spatial and physicochemical properties of functionally important protein regions during the evolution of young chimeric genes^14^. Three retrogenes in *Drosophlia* species (i.e., *Jingwei*, *Adh-Finnegan* and *Adh-Twain*) underwent a rapid adaptive amino acid evolution in the encoded amino acid sequence after they were formed, which was then followed a period of quiescence and functional constraint^15,16^. This pattern of change represents the first insight into the adaptive evolutionary process of the new genes.

Although chimeric retrogenes are generated in rice genomes at relatively high rates, the patterns in the sequence evolution and the mechanisms underlying the development of these new retrogenes remain unclear. To clarify these two critical aspects of the evolution of new genes, we designed primers specific for more than a hundred randomly selected new genes from 380 previously identified chimeric retrogenes^13^. After gel electrophoresis, 24 retrogenes were chosen based on their high-quality polymerase chain reaction (PCR) results. The resulting data for the retrogenes and their parental genes enabled an investigation of the evolutionary patterns of rice retrogene pairs and an examination regarding whether chimeric gene are under positive selection soon after retrogenes form.

## Results

### Seven retrogene pairs undergo positive selection

According to the results of three methods, we determined that 7 of 24 retrogene pairs are under positive selection. All the log likelihood (lnL) values and the parameters of the final optimal models for seven retrogene pairs for each method are shown in Table 1. Details regarding the other 17 retrogenes are provided in Table S1. This information laid the foundations for analyses of specific sites (Table 2). All these analyses are described in detail as follows.

**Table 1:** Log likelihood values of seven chimeric retrogene pairs.

	OBSM method	ORM (lnL value)	Final Optimal model	Free-Model
*AK070196*(*RCG1*)	Method I	−1001.441743 (np = 14)	−999.367951 (np = 15)	−995.133891 (np = 25)
	Method II			
	Method III		−996.78059 (np = 15)	
AK106715(*RCG2*)	Method I	−1385.374644 (np = 14)	−1381.523869 (np = 15)	−1377.501566 (np = 25)
	Method II			
	Method III		−1380.484048 (np = 15)	
AK072107 (*RCG3*)	Method I	−2108.544224 (np = 18)	−2105.905565 (np = 19)	−2101.002768 (np = 33)
	Method II			
	Method III		−2104.405182 (np = 19)	
AK102855 (*RCG4*)	Method I	−2638.742070 (np = 32)	−2595.790736 (np = 38)	−2580.376384 (np = 61)
	Method II		−2587.666653 (np = 37)	
	Method III		−2586.485566 (np = 34)	
AK105722 (*RCG5*)	Method I	−1525.257954 (np = 18)	−1523.006910 (np = 19)	−1517.473148 (np = 33)
	Method II			
	Method III		−1520.804793 (np = 19)	
AK107097 (*RCG6*)	Method I	−519.622517 (np = 8)	−508.323754 (np = 9)	−508.196430 (np = 13)
	Method II			
	Method III			
AK064639 (*RCG7*)	Method I	−1086.356427 (np = 22)	−1058.334507 (np = 27)	−1054.066396 (np = 41)
	Method II		−1058.527587 (np = 26)	
	Method III		−1058.418009 (np = 24)	

ORM, one ratio model; OBSM, optimal branch-specific model.

**Table 2:** Branch-site model-based estimation of seven chimeric retrogene pairs.

	MA	Fixed_MA	M1a	Test 1 df = 2 (MA vs M1a)	Test 2 df = 1 (MA vs Fix_MA)	ω ratio	Parameter estimates	Positively selected sites
RCG1	−989.46	−993.85	−995.55	0.0023	0.0031	ω_0_ = 0.009, ω_2_ = 999	p_0_ = 0.645, p_1_ = 0.153, p_2_ = 0.163, p_3_ = 0.039	1S, 43D, 130P, 138A, 152L
RCG2	−1370.10	−1379.50	−1382.71	3.327e-006	1.453e-005	ω_0_ = 0, ω_2_ = 3.485	p_0_ = 0.364, p_1_ = 0.123, p_2_ = 0.384, p_3_ = 0.129	19S, 29L, 56E, 67G, 68D, 71S, 73I, 74F, 88S, 97G, 127K, 158R, 160Y, 163D
RCG3	−2055.13	−2091.04	−2092.78	P < 0.001	P < 0.001	ω_0_ = 0, ω_2_ = 669.88	p_0_ = 0.461, p_1_ = 0.467, p_2_ = 0.036, p_3_ = 0.036	210G, 211K, 215L, 216N, 218T, 220L, 221E, 228N, 229N, 230F
RCG4	−2562.20	−2563.72	−2608.32	P < 0.001	0.0819	ω_0_ = 0.023, ω_2_ = 1.801	p_0_ = 0.249, p_1_ = 0.084, p_2_ = 0.499, p_3_ = 0.168	3R, 6W, 12A, 26V, 28Q, 40M, 50P, 52N, 54P, 56E, 57I, 58I, 59E, 62I, 65D, 77Q, 78R, 79A, 81Y, 84I, 100P, 107F, 110L, 111L, 116Q, 121A, 122T, 123A, 125G, 127A, 136S, 142R, 144D, 153K, 155S, 156G, 159Q, 164E, 170R, 172V
RCG5	−1491.98	−1497.84	−1497.98	6.182e-004	2.462e-003	ω_0_ = 0.120, ω_2_ = 16.916	p_0_ = 0.602, p_1_ = 0.290, p_2_ = 0.073, p_3_ = 0.035	51Y, 75R
RCG6	−503.11	−508.34	−511.42	2.461e-004	1.218e-003	ω_0_ = 0.004, ω_2_ = 999	p_0_ = 0.925, p_1_ = 0.000, p_2_ = 0.075, p_3_ = 0.000	6G, 7R,8R
RCG7	−1072.84	−1073.88	−1077.28	0.012	0.149	ω_0_ = 0.066, ω_2_ = 12.808	p_0_ = 0.788, p_1_ = 0.061, p_2_ = 0.140, p_3_ = 0.01	18L, 28G, 40G, 48S, 76V

MA, model A of branch-site model analysis in PAML.

#### RCG1

*RCG1* is a new gene that originated 3.15 million years ago (Ks ≈ 0.041) in the rice genome. The log likelihood (lnL) value of the optimal model of method III is −996.78, is significantly better than the lnL value of the optimal model of the method I and method II (LRT: df = 1 2ΔL = 5.17 p-value = 0.023). This result indicates that method III more suitable for *RCG1* data. The estimating of Ka/Ks ratio of lineage branch 9 in the final optimal model of the method I and method II were infinite (999), and the Ka/Ks ratio of branch 9, 8, 11 and 5 in the final optimal model of method III is infinite (999). All these models indicate that the evolution pattern of *RCG1* retrogene pair is episodic. Although it failed in likelihood ratio test (LRT: df = 1, 2ΔL = 3.006, p-value = 0.083) when we nested a comparison between the final optimal model and fix-model which fixed the Ka/Ks ratio of branch 9, 8, 11 and 5 to one, the estimates of parameters in this optimal model suggest that there are sixteen non-synonymous substitutions versus zero synonymous substitution occurred along the lineage 8, it has a high possibility that lineage 8 is undergoing positive selection that the previous study suggested positive selection when the non-synonymous substitutions are greater than 9 while the synonymous substitution is equal to 0 (Nozawa *et al*. 2009). Based on the final optimal model of method III, we used the branch-site model to identify the positive sites. In test 1, M1a (lnL = −995.55) versus Model A (lnL = −989.46), 2Δl = 12.17, p-value = 0.0023 (df = 2); in test 2, Model A versus fix-Model A (lnL = −993.85), 2Δl = 8.77, p-value = 0.0031 (df = 1). All these two tests indicate that the Model A fit the data better than others, Model A suggests five sites to be potentially under positive selection along the foreground branch at the 95% level according to the BEB analysis, these sites are 1 S, 43D, 130 P, 138 A, 152 L, the parameters estimate by Model A are p0 = 0.645, p1 = 0.153, p2 = 0.163, p3 = 0.039, ω0 = 0.009, ω2 = 999.

#### RCG2

*RCG2* is a new gene that originated 6.92 MYA (Ks ≈ 0.090) in the rice genome. The OBSM methods suggest that, excepting lineage 4 in final optimal model of the method I and method II, lineage 4 *Nivara* a and b_P and lineage 1 *Indica-Japonica* P&C in final optimal model of the method III. The Ka/Ks ratio is less than 1 (0.358, 0.321 respectively), all other lineages are greater than 1 (1.744, 1.835 respectively). The log likelihood (lnL) values of these two models are −1381.52 and −1380.48, respectively. Since they have the same ω ratio numbers, the latter model is considered being better because of lower lnL value. That the *RCG2* retrogene pair were undergoing positive selection is confirmed when we nested a comparison between the fix-model and corresponding final optimal models, the 2ΔL is 6.474, the p-value is 0.011. The final optimal model indicates that the positive selection permeates the whole evolution pattern of *RCG2* retrogene pair. The estimates of parameters in the final optimal models suggest that the non-synonymous substitutions in five lineages 3, 7, 5, 6 and 2 are all greater than 9, range from 10.5 to 26.3.

Model A is more suitable than others based on the final optimal model, two branch-sites model tests. Nine sites were identified to be potentially under positive selection along the foreground branch at the 95% level according to the BEB analysis (19S, 29L, 56E, 67G, 68D, 71S, 73I, 74F, 88S, 97G, 127K, 158R, 160Y, 163D). The parameters suggested by Model A are p0 = 0.364, p1 = 0.123, p2 = 0.384, p3 = 0.129, ω0 = 0, ω2 = 3.485.

#### RCG3

*RCG3* is homologous to a *Verticillium wilt* resistance gene *Ve1*^17,18^ which originated 14.77 MYA (Ks ≈ 0.192) in the rice genome. The lnL value of final optimal model of the method I and method II is −2105.91, the lnL value of the final optimal model of method III is −2104.41. Since they have the same ω ratio numbers, the latter model was chosen. The estimate of Ka/Ks ratio of lineage *Nivara* b_P in final optimal model of the method I and method II is 1.388, the estimate of Ka/Ks ratio of branch 15, 6 and 10 in the final optimal model of method III is 1.524. Although these two models do not show significance in LRTs tests, when we nested a comparison between the fix-model and final optimal model, it is suggested that branch 6 has a much higher substitution rate than the background substitution rate due to the large number of non-synonymous substitutions (30.3 and 31.0 respectively).

Based on the final optimal model, using the two branch-sites model tests based on the final optimal models indicates that Model A fits the data best. Model A suggests ten sites to be potentially under positive selection along the foreground branch at the 95% level according to the BEB analysis, these sites are 210G, 211K, 215L, 216N, 218T, 220L, 221E, 228N, 229N, 230F. Surprisingly, all these sites are nearly adjacent and seem to comprise a functional unit. The parameters suggested by Model A are p0 = 0.461, p1 = 0.467, p2 = 0.036, p3 = 0.036, ω0 = 0, ω2 = 669.88.

#### RCG4

Given the complexity of the sixteen sequences included in this retrogene pair, the result of the estimating models suggested by OBSM are inconclusive. The final optimal model suggested by Method I is a seven-ratio model and the lnL value is −2595.79. The final optimal model suggested by Method II is a six-ratio model and the lnL value is −2587.67. The final optimal model suggested by Method III is a three-ratio model and the lnL value is −2586.49. Obviously, the final optimal model of Method III fit the data better than other two models since the fewer parameters and the larger lnL value. Although this model failed in LRTs when we nested a comparison between the fix-model and final optimal model, it is suggested by all three final optimal models that the lineage *Nivara* b_P have a much higher substitution rate than the background substitution rate. The estimates of parameters in these three optimal models suggest that the non-synonymous substitutions in lineage *Nivara* b_P are 18.7, 18.7 and 16.5 respectively.

Based on the final optimal model of method III, two tests indicate that the Model A fit the data better than other models. Model A suggests many sites to be potentially under positive selection along the foreground branch at the 95% level according to the BEB analysis. The parameters suggested by Model A are p0 = 0.249, p1 = 0.0084, p2 = 0.499, p3 = 0.168, ω0 = 0.023, ω2 = 1.801.

#### RCG5

The lnL value of the final optimal model of Method I and Method II is −1523.01, the lnL value of final optimal model of Method III is −1520.80, the latter one is significantly better than the former one according to the LRTs (df = 1, 2ΔL = 4.404, p-value = 0.036). This result indicates that the final optimal model of method III fit *RCG5* gene pair better than the former model. The estimating of Ka/Ks ratio of lineage Glab_P in final optimal model of method I and method II is 2.20, and the estimating of Ka/Ks ratio of lineage Glab_P, branch 10, and lineage *Nivara* a in the final optimal model of method III is 2.66. All these models indicate that the evolution pattern of RCG5 retrogene pair is episodic. Although it failed in LRTs (df = 1, 2ΔL = 2.612, p-value = 0.106) when we nested a comparison between the final optimal model and fix-model which fixed the Ka/Ks ratio of lineages Glab_P, branch 10 and Nivara-a equals to one. The estimates of parameters in final optimal model of method III suggest that there are about 10.8 non-synonymous substitutions along the branch 10, and there’re 16.6 non-synonymous substitutions along the lineage Glab_P, it has a great possibility that the branch 10 and Glab_P are undergoing positive selection.

Based on the final optimal model of method III, we used branch-site model to identify the positive sites. In test 1, M1a (lnL = −1497.98) versus Model A (lnL = −1491.98), 2Δl = 12.00, p-value = 0.0025 (df = 2), in test 2, Model A versus fix-Model A (lnL = −1497.84), 2Δl = 11.72, p-value = 0.0006 (df = 1). All these two tests indicate that the Model A fits the data better than others, Model A suggests two sites to be potentially under positive selection along the foreground branch at the 95% level according the BEB analysis, these sites are 51Y, 75 R, the parameters suggested by Model A are p0 = 0.602, p1 = 0.290, p2 = 0.073, p3 = 0.035, ω0 = 0.121, ω2 = 16.92.

#### RCG6

The three OBSM methods suggested an identical final optimal model. The estimating of Ka/Ks ratio except branch 5 is suggested to be infinite (999). Although it failed in LRTs (df = 1 2ΔL = 3.108 p-value = 0.0779) when we nested a comparison between the final optimal model and fix-model which fixed the Ka/Ks ratio of all lineages equal to one except branch 5, the estimates of parameters in this optimal model suggest that they are about 19.5 non-synonymous substitutions versus 7.1 synonymous substitutions occurred along the branch 5, it has a great possibility that the lineage B is undergoing positive selection.

Based on the final optimal model, we used the branch-site model to identify the positive sites. In test 1, M1a (lnL = −511.42) versus Model A (lnL = −503.11), 2Δl = 16.62, p-value = 2.461e-004 (df = 2), in test 2, Model A versus fix-Model A (lnL = −508.34), 2Δl = 10.46, p-value = 1.218e-003 (df = 1). All these two tests indicate that the Model A fit the data better than others, Model A suggests three sites to be potentially under positive selection along the foreground branch at the 95% level according to BEB analysis, these sites are 6G, 7R, 8R, the parameters suggested by Model A are p0 = 0.925, p1 = 0.00, p2 = 0.0753, p3 = 0.00, ω0 = 0.0045, ω2 = 999.

#### RCG7

Given the complexity of these eleven sequences included in this retrogene pair, the result of the most probable estimating models suggested by OBSM are all different. The final optimal model suggested by Method I is a six-ratio model and the lnL value is −1058.33. The final optimal model suggested by Method II is a five-ratio model and the lnL value is −1058.53. The final optimal model suggested by Method III is three-ratio model and the lnL value is −1058.42. Although the final optimal model of the Method III has fewer parameters than other two models, the lnL value of these three models are very close to each other. This final optimal model of Method III suggested the Ka/Ks ratios of all lineages are less than one while other two models all suggested the branch 18 and lineage *Grandi_P* are larger than one. Although all LRTs comparisons between the final optimal models of Method I and Method II and fix-model in which fix branch 18 and lineage *Grandi_P* equal to one failed, it is suggested by the two final optimal models that the branch 18 have a much higher substitution rate than the background substitution rate since the estimates of parameters suggest that there are 7.6 non-synonymous substitutions versus 1.1 synonymous substitutions occurred along the branch 18.

We used the branch-site model to identify the positive sites, with suggested test 1 and 2 detected positive selection sites along branch 18. Test 1 suggested that Model A is significantly better than the model M1a while it failed in test 2. Model A suggests five sites to be potentially under positive selection along the foreground branch at the 95% level according to the BEB analysis; these sites are 18L, 28G, 40G, 48S, 76V. The parameters suggested by Model A are p0 = 0.788, p1 = 0.0612, p2 = 0.140, p3 = 0.0109, ω0 = 0.0662, ω2 = 12.81.

### Tajima’ D test suggests the mutations in RCG4, RCG6 are deviation from neutral mutation hypothesis

To address whether retrogenes are under neutral selection, we used Tajima’ D test in MEGA 7 to examine chimeric retrogene mutations^19^. Significant results were obtained only for *RCG4* and *RCG6* pairs. Specifically, Tajima’ D deviated significantly from 0 for *RCG4* (*p* < 0.01) and *RCG6* (p < 0.001) (Table 3).

**Table 3:** Results of Tajima’s neutrality Test for chimeric retrogene pairs.

	m	S	p_s_	Θ	π	D
*RCG4*	16	313	0.570	0.172	0.270	2.486
*RCG6*	4	79	0.357	0.195	0.240	2.443

The Tajima test statistic was estimated with MEGA7. All positions containing gaps and missing data were eliminated from the dataset (i.e., complete deletion option). m = number of sites; S = number of segregating sites; ps = S/m; Θ = ps/a1; π = nucleotide diversity; D = Tajima test statistic.

### Substitution patterns in the new retrogenes and in the parental genes

**Figure 1:**
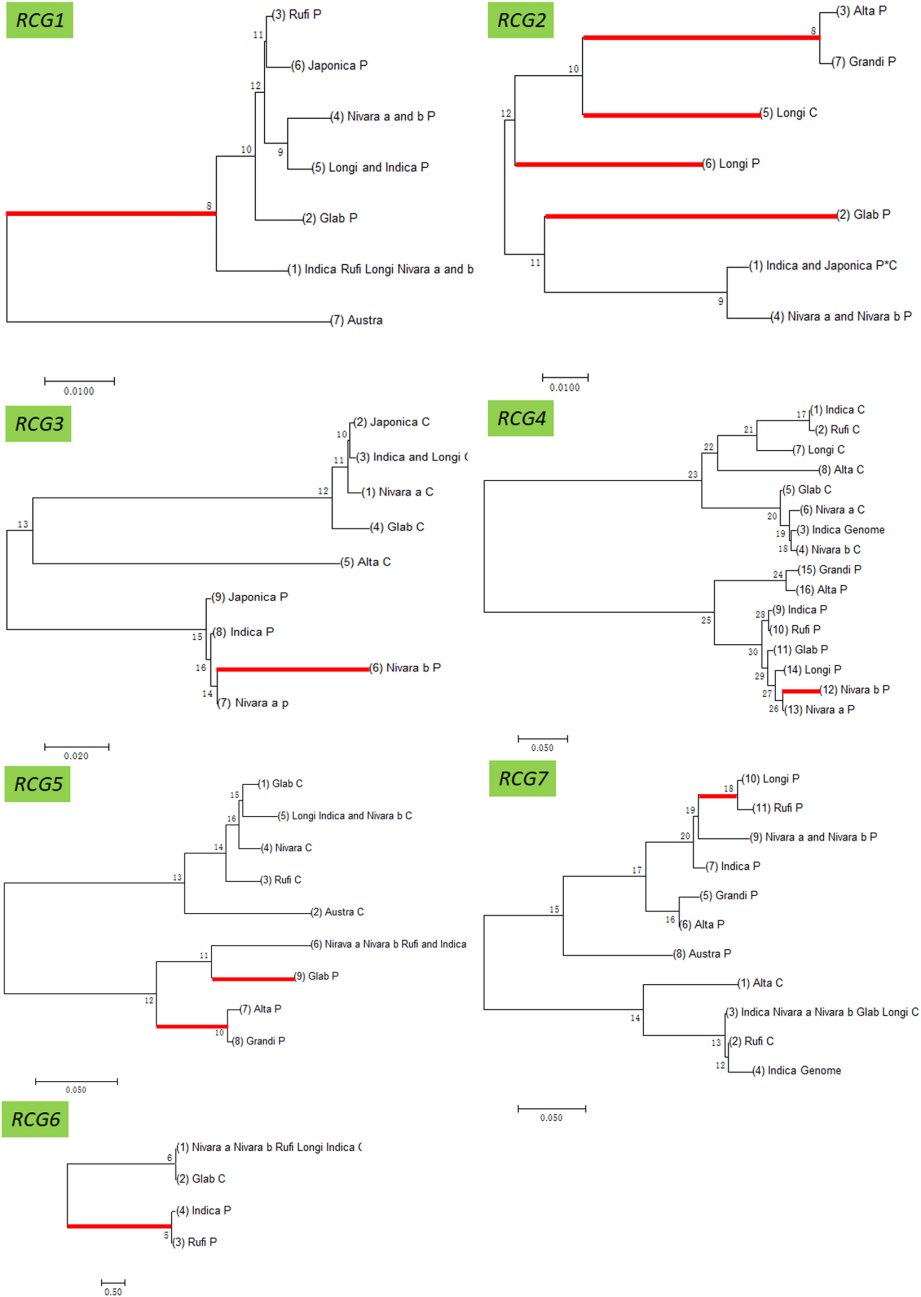
Phylogeny of seven chimeric retrogene pairs. A phylogenetic tree was constructed with the default parameters of MEGA7; P and C represent the parental and chimeric retrogene sequences, respectively; Genome is added to the end of species names when the corresponding genomic region of *Indica* (9311) was used instead of *RCG* sequences that were amplified by PCR in sibling species, but not in 9311. Positive selection is indicated with a red branch. The species names are the same as the abbreviated names in Table S2.

Three distinct patterns were detected based on synonymous substitution and replacement sites in seven gene pairs (Fig. 1). In Pattern 1, the chimeric genes were rapidly substituted in the initial stage of the new gene lineage under positive selection (e.g. *RCG2*). This is somewhat consistent with a previously described pattern (Jones and Begun 2005; Jones *et al*. 2005), in which three new *Adh*-related retrogenes evolved rapidly after the new genes were formed. Furthermore, our results implied the parental gene also rapidly evolved. Several instances of this type of rerouted functional evolution were observed. In Pattern 2, the parental genes evolved rapidly soon after the chimeric genes were formed, whereas the new genes evolved slowly (e.g., *RCG6*). This pattern was reflected by the pseudogenization of the parental copy of a *mkg-p* gene in *D. mauritiana*^5^. In Pattern 3, the parental genes evolved after the chimeric genes were formed, but only after some time had passed, and the new genes evolved slowly (e.g., *RCG3, RCG4, RCG5* and *RCG7*). Pattern 2 and 3 implied that the parental gene functionality evolved via an unexpected process. To avid functional redundancy of retro copies, the new retrogenes might have replaced the parental gene to complete the ancestral functions, while the parental gene neo-functionalized because of adaptive evolution.

### *RCG3* may be important for disease resistance

**Figure 2:**
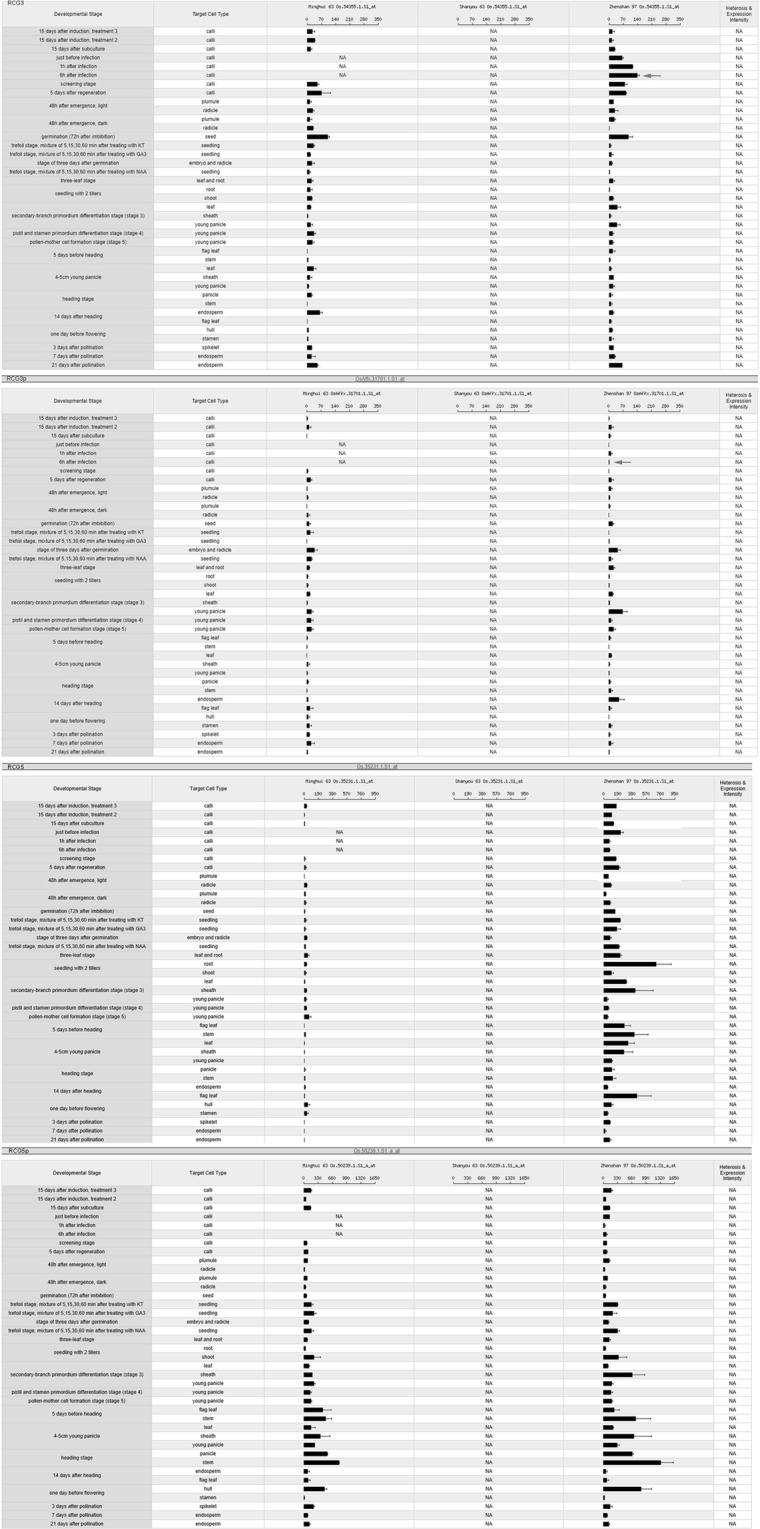
Diversity in the expression of *RCG3* and *RCG5* and their parental genes. The corresponding sequences were used as queries to search the Affymetrix Rice Genome Array data. Digital expression profiles were generated with an online tool (http://crep.ncpgr.cn/crep-cgi/blast.pl). For the chimeric retrogenes, red arrows indicate the stage during which the expression level was highest. The corresponding stage for the parental gene is indicated with red arrows.

We compared our seven chimeric retrogenes to the probe sets of Affymetrix GeneChip Rice Genome Arrays because of the high complexity and the redundancy of the retrogenes (Table 4) as well as the incomplete probe set coverage of the rice genome. Only the *RCG3* and *RCG5* pairs had a perfect match to a probe set (Table 5). Expression profiles were obtained from the CERP database (http://crep.ncpgr.cn/). However, both *RCG3* and *RCG5* exhibited functional divergence (Fig. 2), especially according to the life cycle of rice gene expression data^20^. Additionally, the expression of the *RCG3* probe (Os.54355.1.S1_at) in Zhenshan 97 (cultivated rice variety) peaked in calli during the infection period, in seed germination period (72 h after imbibition), and in the endosperm at 21 days after pollination. These results were consistent with the independent evidence in the TIGR (http://rice.plantbiology.msu.edu), where this gene encodes Leucine-rich protein, and is highly similar to the *Ve1* gene conferring resistance to *Verticillium wilt* disease (Fradin *et al*. 2009; Kawchuk *et al*. 2001).

**Table 4:** Copy number variations for the similarity hits in the OMAP/OGE genomes.

	RCG1	RCG2	RCG3	RCG4	RCG5	RCG6	RCG7	Genome_size (Mb)
*Oryza barthii*	118	106	12000	59	104	39	794	760
*Oryza brachyantha*	10	55	6667	47	28	4	729	389
*Oryza glaberrima*	131	102	11609	60	90	30	1240	389
*Oryza longistaminata*	89	214	806	129	95	48	217	760
*Oryza meridionalis*	161	36	11201	70	80	34	298	760
*Oryza nivara*	136	54	12000	81	90	36	821	539
*Oryza punctata*	1148	38	9116	96	58	13	1776	1691
*Oryza rufipogon*	160	79	12000	120	122	37	1315	1201
*Oryza sativa indica*	146	84	12107	155	124	29	1382	1000
*Oryza sativa japonica*	142	67	12037	158	125	35	1678	1054

Genome sequences of seven *RCG* genes were used as queries for blastn searches of the Gramene database (e-value threshold of 1e-5).

**Table 5:** Affymetrix GeneChip expression profiles of seven chimeric retrogene pairs.

	Chimeric retrogene ID in Plant cell paper	Chimeric Affy Probset names	Parental Affy Probset names
*RCG1*	Chr03_4107, AK070196_Chr03_27608263_27613159	NA	NA
*RCG2*	Chr04_4524, updata_AK106715_Chr04_30664045_30669070	Os.57563.1.S1_at	NA
*RCG3*	Chr12_904, updata_AK072107_Chr12_5820378_5826726	Os.54355.1.S1_at	OsAffx.31701.1.S1_at
*RCG4*	Chr10_2602, updata_AK102855_Chr10_17747411_17752061	NA	OsAffx.29724.1.S1_at
*RCG5*	Chr01_5436, updata_AK105722_Chr01_36521616_36526443	Os.35231.1.S1_at	Os.50239.1.S1_a_at
*RCG6*	Chr02_1920, updata_AK107097_Chr02_12785386_12789823	NA	Os.54261.S1_at
*RCG7*	Chr08_3454, updata_AK064639_Chr08_24470676_24475311	NA	NA

Chimeric and parental gene sequences were used to search the CREP rice expression profile database (http://crep.ncpgr.cn/crep-cgi/home.pl). A probe was applied to a target sequence only when there were no mismatches (e-value = 0) and were hybridised to the right position. NA, no perfect match for chimeric retrogene pairs.

### Chimeric retrogenes have more recent origination

The following Ks values were calculated for *RCG1-7* based on a two sequence comparison (i.e., parental *vs* new genes): 0.124, 0.19, 0.281, 2.27, 0.547, 1.884 and 3.575, respectively. Because of the increased availability of sequence data, we recalculated the Ks value for *RCG1, RCG2* and *RCG3* according to the NG86 model of MEGA7^21,22^, with the transition/transversion ratio k = 2. To accurately estimate the divergence time, the *Oryza australiensis* branch (Fig. 1) was excluded from the *RCG1* data during the analysis because it is ancestral to the clade generated by the retroposition event. The Ks values with a 95% confidence interval for *RCG1*, *RCG2* and *RCG3* are 0.041 ± 0.011, 0.090 ± 0.016 and 0.192 ± 0.021 respectively. Assuming that the synonymous substitution rate of rice genes is 6.5 × 10^−9^ substitutions per site per year^23^, then these chimeric retrogenes would have formed approximately 3.15 ± 0.88 MYA, 6.92 ± 1.23 MYA and 14.77 ± 1.62 MYA, respectively. These estimates implied these three chimeric retrogenes are very young (*RCG1* and *RCG2*) or young (*RCG3*).

## Discussion

In this study, we used the Optimal Branch Specific Model (OBSM) program (Zhang *et al*. 2011) to analyze chimeric genes based on the optimal branch model. Specifically, OBSM is a CODEML program of the PAML package^24^ designed to help the user to select optimal branch-specific models^25^ with a maximum likelihood approach. We also used the branch site approach to explore sites under positive selection even though this method has some disadvantages. For example, it may not suggest correct sites proposed by Nozawa, *et al*.^26^. In fact, in our data analysis, especially regarding *RCG3*, the sites suggested by the MA model seemed reasonable because these all belong to leucine-rich repeat regions potentially related to disease resistance. Individuals resistant to diseases may be more likely to be selected than those susceptible to diseases.

Common patterns and mechanisms underlying the evolution of new genes were previously generalized in many studies. For example, Corbin D. Jones (Jones and Begun 2005; Jones *et al*. 2005) analyzed the origins of *Jingwei*, *Adh-Finnegan*, and *Adh-Twain* in *Drosophila* species, and confirmed the genes underwent a rapid adaptive evolution affecting the amino acid sequence shortly after they were formed. This initial period of rapid change was followed by quiescence and functional constraint. In 2008, a study of novel alcohol dehydrogenases (*siren1* and *siren2*) indicated that chimeric genes evolved adaptively shortly after they were formed^27^. However, our results revealed another pattern. Specifically, in addition to the rapid adaptive evolution of chimeric retrogenes soon after they were formed, the parental genes also underwent a rapid adaptive evolution. This evolution of the parental genes was observed for six of the analysed chimeric retrogene pairs (*RCG2* to *RCG7*). Differences between *Drosophila* and *Oryza* species may have been due to the high proportion of retrotransposons in rice^28–30^ or because of a recent segmental duplication event that approximately 5 MYA^31^ and high gene reshuffling in rice genome^32^. Subsequent large-scale chromosomal rearrangements and deletions may have influenced the evolution of the chimeric retrogene pairs.

To compare the expression profile of *RCG3* and its parental gene, we located the *RCG3* parental gene in the *Oryza Sativa L. japonica* genome. According to the TIGR database, the predicted parental gene locus is LOC_Os12g11370. The probe set (OsAffx.31701.1.S1_at) for this region revealed that the parental gene is most highly expressed in young panicles during the secondary branch primordium- differentiation stage (stage 3) (Fig. 2), whereas only a negligible signal was detected for the parental gene at this stage. This observation may be explained by the fact that a high expression level in a generative organ may lead to retrotransposition in the genome^33^.

In this study, 7 of 24 (29.17%) chimeric retrogene pairs were identified as being under positive selection. This proportion is much higher than that revealed during a whole-genome analysis of *Streptococcus*^34^ and *Apis mellifera*^35^. A phylogenomic analysis of *Streptococcus*^34^ proved that 136 of 1730 gene clusters (7.86%) underwent positive selection. A genome-wide analysis of positive selection in *A. mellifera* (honey bee) suggested that positive selection affected at least 852–1,371 genes, corresponding to about 10% of the bee’s coding genome^35^. If we assume that, on average, 10% of the coding genes in a genome are under positive selection, then the 29.17% of the chimeric retrogenes under positive selection is significantly higher according to the Fisher exact test (p = 0.001). We speculated that reverse transcribed mRNA intermediates confer new chimeric retrogene pairs with advantages for survival or propagation.

## Methods

### Samples, primers and molecular cloning

Ten species and two subspecies were included in this study. The following seven species were obtained from the International Rice Research Institute (the International Rice Germplasm Collection ID numbers are provided in Table S2): *Oryza grandiglumis* (shortened to *Grandi*), *Oryza longistaminata* (*Longi*), *Oryza alta* (*Alta*), *Oryza australiensis* (*Austra*), *Oryza rufipogon* (*Rufi*), *Oryza nivara* (*Nivara a* and *b*), and *Oryza glaberrima* (*Glab*). The other two species, *O. punctate* (*YSD8)* and *O. officinalis* (OWR) were provided by Shiping Wang’s laboratory. The genomes of two subspecies, *Oryza sativa* L*. indica* (*Indica*) and *Oryza sativa* L*. japonica* (*Japonica*), were used as fully sequenced reference genomes. Total genomic DNA was isolated from leaves according to the Cetyl Ttrimethyl Ammonium Bromide (CTAB) method. Genomic DNA for YSDB (BB genome) and OWR (CC genome) was obtained from Wang laboratory.

All primers were designed according to the *Oryza sativa* L. *japonica* and *Oryza sativa* L. *indica* genome sequences (Table S3; the other 17 primer pairs are not provided). Because of the extreme sequence redundancy around the chimeric retrogenes regions, the primers targeted the flanking sequences for a PCR amplification of approximately 1-kb amplicons, which were sequenced from the 5′ ends with the ABI Prism 3730 sequencer (Applied Biosystems, Foster City, CA, USA). All of the sequences included in our study were derived from PCR sequencing, except in cases where the PCR did not amplify the reference genome sequences, but did amplify the genome sequences of *Indica* accession 9311. In these cases, the *Indica* 9311 genome sequence was used instead of the *Oryza sativa L. indica* genome sequence for subsequent analyses.

### Sequencing region details

In a previous study^13^, an analysis of the 898 intact retrogenes identified in *Indica* (9311) during an *in-silico* analysis indicated that 380 retrogenes have chimeric structures. From these 380 retrogenes, we sequenced 24, of which, seven on certain specific branch were under positive selection. These seven retrogenes are *RCG1* (retrochimeric gene 1, chimeric id Chr03_4107; the chimeric id was consistent with the data in 2006 paper^13^), *RCG2* (Chr04_4524), *RCG3* (Chr12_934), *RCG4* (Chr10_2602), *RCG5* (Chr01_5436), *RCG6* (Chr02_1920), *RCG7* (Chr08_3454). To exclude the artifacts of genome sequencing and assembly in 9311, we used these seven chimeric retrogenes and their parental genes as queries to screen the new PacBio genome IR8 (Table S4). According to a previous study and the information in a publicly accessible database (Gramene), these seven retrogenes lack similar chimeric homologs in maize and sorghum. The chimeric structure of three retrogenes is demonstrated in Fig. S1.

### Sequence edit and blast analysis

We cloned sequences from wild rice genomic DNA with the designed primers. The amplicon sequence statistics are listed in Table S5. During the computational evolutionary analysis, the sequences cloned by PCR that were not long enough or could not be aligned with a retrogene were eliminated. Regarding *RCG4* and *RCG7*, the *Oryza sativa* L. *indica* sequence (Indica in Fig. 1) was highly similar to the reference genome sequence (Indica Genome in Fig. 1), and we were unable to confirm which one is orthologous to sequences in other species. Consequently, both the PCR sequence and genome sequence were used for the calculation in this study.

### Molecular evolution analysis

#### Phylogenetic reconstruction

**Figure 3:**
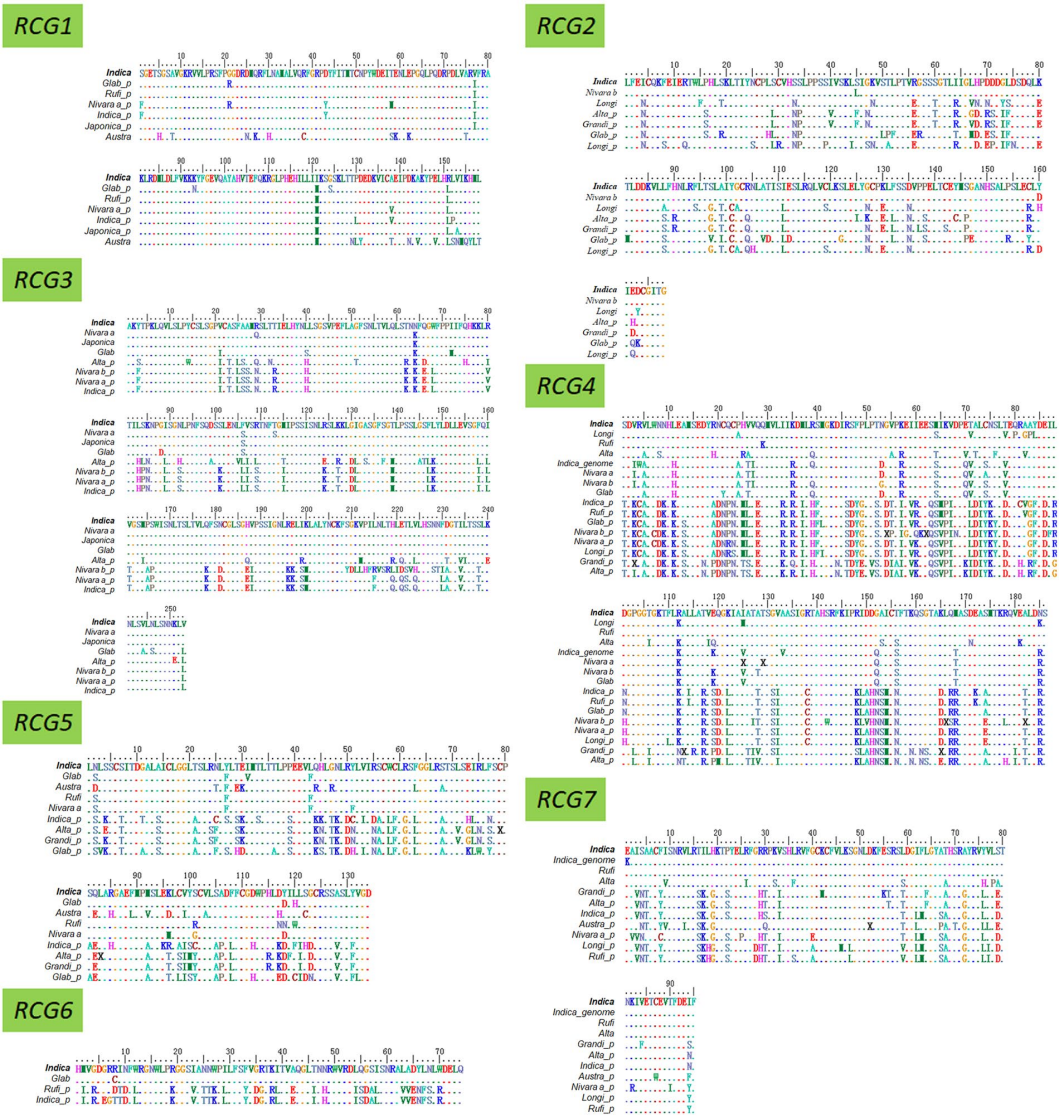
Alignment of the amino acid sequences encoded by seven chimeric retrogene pairs; *_p* represents the parental gene sequence; _*genome* indicates the corresponding genomic region of *Indica* (9311) was used in place of an *RCG* that was amplified by PCR in sibling species, but not in 9311, or produced an amplicon that differed from that of 9311. Dots signify the amino acid at that particular position is the same as that in the 9311 sequence. The species names are the same as the abbreviated names in Table S2.

The sequences of the coding regions the retrogene pairs were first translated to amino acid sequences based on the chimeric retrogene structure according to the reference sequences. After a sequence alignment with the ClustalW program of MEGA7^36^, the amino acid sequences were reconverted to nucleotide sequences. The alignments of the amino acid sequences encoded by the seven candidate retrogene pairs under positive selection and the other 17 retrogene pairs are presented in Figs. 3 and S2, respectively. Phylogenetic relationships were determined with the default parameter of neighbor-joining methods of MEGA7. The phylogenetic tree among the seven retrogene pairs under positive selection and the other 17 retrogene pairs are provided in Figs. 1 and S3, respectively.

#### Maximum likelihood analysis for estimating the parameters

We used the OBSM (Optimal Branch Specific Model) program^37^ to identify the most appropriate branch-specific model for estimating the number of non-synonymous substitution per non-synonymous site (Ka) and the number of synonymous substitutions per synonymous site (Ks) respectively as well as the corresponding omega (ω = Ka/Ks) ratio. Additionally, ω > 1, suggests positive selection; whereas ω ≈ 1 suggests neutral evolution and ω < 1 suggests purifying selection with a functional constraint. The OBSM program comprises three methods. The first method can be completed relatively quick, whereas the second and third method is more time-consuming, but produces a better result for a more ideal branch-specific model in the likelihood ratio test (LRT)^37^ or the Akaike Information Criterion (AIC) comparison^38^.

We analyzed all these 24 retrogene sets with the three methods of OBSM program. During the analysis, we removed all gaps in alignments, set the codon frequency of the CODEML control file at CodonFreq = 3, and set the parameter k in method 3 of OBSM at 0.5. Furthermore, we used the branch-site model^39^ to explore the positive selection sites and fix the specific branch identified by the final optimal models as the foreground branch. The suggested tests 1 and 2 were employed for detecting positive selection sites^40^.

## Supplementary information

Supplemental Informations

Table S1

## Data Availability

All data and resources in the manuscript are available upon reasonable request to the corresponding authors.
