# Supplementary material for: Evolutionary patterns of chimeric retrogenes in Oryza species
Source: Sci Rep. 2019 Nov 27;9:17733. doi: 10.1038/s41598-019-54085-2 (PMC6881317; doi:10.1038/s41598-019-54085-2)
Supplement: Supplementary file 1 — Supplemental Informations [file 41598_2019_54085_MOESM1_ESM.pdf]

Evolutionary patterns of chimeric retrogenes in *Oryza* species

Yanli Zhou[1], Chengjun Zhang[1,2*]

[1]Germplasm Bank of Wild species, Kunming Institute of Botany, Chinese Academy of Sciences. No. 132 Lanhei Road, Kunming 650201, Yunnan, China

[2]Haiyan Engineering & Technology Center, Kunming Institute of Botany, Chinese Academy of Science. Jiaxing 314300, Zhejiang, China

*Correspondence author: Chengjun Zhang; email: zhangchengjun@mail.kib.ac.cn

Table S2. Species analysed in this study

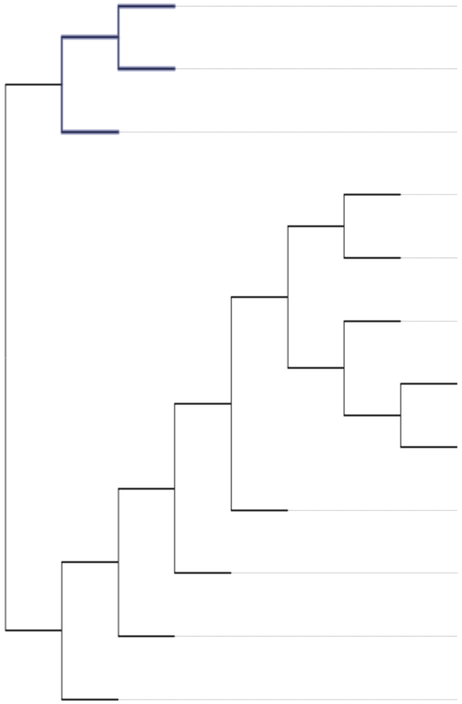

ID	Species/subspecies	Short Name	Genome	IRGC ACC
1	*Oryza grandiglumis (Doell Prod.)*	*Grandi*	CCDD	105664
2	*Oryza alta (Swallen)*	*Alta*	CCDD	100967
3	*Oryza officinalis*	*OWR*	CC	/
4	*Oryza Sativa L. japonica*	*Japonica*	AA	/
5	*Oryza rufipogon (Griff.)*	*Rufi*	AA	80643
6	*Oryza Sativa L. indica*	*Indica*	AA	/
7	*Oryza nivara (Sharma et Shastry)*	*Nivara a*	AA	80622
8	*Oryza nivara (Sharma et Shastry)*	*Nivara b*	AA	80582
9	*Oryza glaberrima (Steud.)*	*Glab*	AA	103600
10	*Oryza longistaminata (A. Chev. et Roehr)*	*Longi*	AA	103886
11	*Oryza punctata*	*YSD8*	BB	/
12	*Oryza australiensis (Domin)*	*Austra*	EE	86530

Note: '/' indicates the genomes of model species (*Oryza sativa* L. *indica* and *Oryza sativa* L. *japonica*) or wild accessions (*Oryza punctata* and *Oryza officinalis*) from the Wang laboratory were used as reference genomes. The phylogenetic tree on the left presents the phylogenetic relationships among the 12 analysed rice species.

Table S3. Details regarding the primers designed for PCR and sequencing

ID		Primer pair	primer	Product (bp)
RCG1	Chimerical retrogene	1	CGAGATTAACATTCTCATC<br>GCTTCAGGTTACAGGTTAG	1260
		2	CAAAACAGCCGGATAGATAC<br>GACATTGTCTCCCATCCGAG	1005
	Parental gene	3	GTCCTAGAAGAAGATGGTCG<br>CCAAGATTCAACAGACAAG	1085
		4	CCAATTATCCAGCGATAGTG<br>GACATTGTCTCCCATCCAAG	1196
RCG2	Chimerical retrogene	1	CTGGAAGGATGCATGGAATGG<br>CTCAGTTCCTGTAGGGCCTG	1094
	Parental gene	2	GAACTCCAGTTTAAAGGTTCTG<br>CACAGCTTCGAATTATCAACTC	1912
		3	CAGGCAGGTGAGGTTTCCTGG<br>CTGAGTTTCTGTTCCTGATGG	1332
RCG3	Chimerical retrogene	1	GCAGCGGTACATATTGATGG<br>ACGCTGTAGACTCCATTGGG	1377
	Parental gene	2	TCATCCAACATGGGAAGGAG<br>ACCTTGACCTCTCCACAAGC	1166
RCG4	Chimerical retrogene	1	TGCCATCCTCACTACGAAGAC<br>TTATGTGGATCGTCTTCCGC	1155
	Parental gene	2	ACATGACGACTGCTTGATCG<br>TCTCTCGTCATCAAGTGAGGG	1181
RCG5	Chimerical retrogene	1	ATTCAAGGAAAGCTGGGTTG<br>GCGAAGAGACGAAGTTACCTG	1170
	Parental gene	2	TGGGAGATCTGGCAAAGATG<br>GCACTGTCATCGGATCATTC	1165
RCG6	Chimerical retrogene	1	GGCACTTCACCGAAGGGTAG<br>GGATCCGTTATCTCCTCTTCC	1087
	Parental gene	2	ATAGCGAGCAGGTCGGTTC<br>ATAACAGCAGGGGCAAAGC	1257
RCG7	Chimerical retrogene	1	GGTTGAAAGGAAGAACCGC<br>TAGCAACTGGTGCAAGGTTTC	966
	Parental gene	2	TTCGTCTCCATGTGTTCCTC<br>ACAGCATTGATCCACGATTC	881

Table S4 Chimerical retrogene and parental gene blat against IR8 genome

	Gene ID in Plant cell paper	Chimeric gene in 9311	Chimeric retrogene in IR8	Parental gene in 9311	Parental gene in IR8
RCG1	Chr03_4107	Chr03:27608262-27613159 (4897)	Chr03: 26049124.. 26054020 (4896)	Chr02:14775401-14780342 (4941)	Chr02: 14335789..4340666 (4877)
RCG2	Chr04_4524	Chr04:30664128-30669070 (4942)	Chr04: 31889774..31894673 (4899)	Chr04:30673408-30679130 (5722)	Chr04: 31713113..31894438 (543826)
RCG3	Chr12_904	Chr12:6133222-6139034 (5813)	Chr12: 5723488..5727995 <4507>	Chr11:19034040-19040398 (6359)	Chr12: 5801686..5807082 (5396)
RCG4	Chr10_2602	Chr10:17747410-17752061 (4652)	Chr10: 20792601..20812983 <3709>	Chr09:4376856-4381567 (4711)	Chr09: 5374301..5378751 (4450)
RCG5	Chr01_5436	Chr01:36521615-36526443 (4828)	Chr01:34682823..34687645 (4822)	Chr05:18541573-18546535 (4962)	Chr05:18326109..18330814 (4705)
RCG6	Chr02_1920	Chr02:12785385-12789823 (4438)	Chr02:12059249..12063688 (4439)	Chr07:9892475-9896854 (4379)	Chr07:10350983..10355365 (4382)
RCG7	Chr08_3454	Chr08:24470675-24475311 (4636)	Chr08:24715472..24720109 (4637)	Chr10:11013463-11018192 (4729)	Chr10:14428416..14432001 (3585)

The sequences of chimeric retrogenes and the corresponding parental genes were used as queries for a blast search of the *Indica* (IR8) rice genome, which was sequenced via PacBio technology. The output of the blast search is provided in round brackets. When the blast search output was too long, the sequences were shortened with gene-specific primers. The resulting data are provided in angle brackets.

Table S5 PCR sequencing of retrogenes and parental genes

	RCG1		*RCG2*		*RCG3*		*RCG4*		*RCG5*		*RCG6*		*RCG7*	
	C	P	C	P	C	P	C	P	C	P	C	P	C	P
Grandi	x	x	x	7	x	x	x	15	x	8	x	na	x	5
Longi	1	5	5	6	3	x	7	14	5	x	1	x	3	10
Alta	x	x	x	3	x	5	8	16	x	7	x	na	1	6
Austra	7	x	x	x	x	x	x	x	2	x	x	x	x	8
Rufi	1	3	x	x	x	x	2	10	3	6	1	3	2	11
Nivara a	1	4	x	4	1	5	6	13	4	6	1	na	3	9
Nivara b	1	4	x	4	x	6	4	12	5	6	1	x	3	9
Glab	na	2	x	2	4	na	5	11	1	9	2	x	3	na
Indica	1	5	1	1	3	8	1&*	9	5	6	1	4*	4&*	7
Japonica	na	6	1	1	2	9	x	x	x	x	x	x	x	x
YSD8	na	x	x	x	x	x	x	x	x	x	x	x	x	x
OWR	x	na	x	x	x	x	x	x	x	x	x	x	x	x
Total number of sequences	7		7		9		16		9		4		9	

C, chimeric retrogene; P: parental gene; x: no amplicon; na: no high-quality sequence was obtained; *: *Indica* reference sequence was used; &: cloned sequence did not exactly match the 9311 reference sequence.

Total number of sequences refers to the number of sequence types used for analysing phylogeny, and corresponds to the maximum value in the C and P columns for each retrogene.

The same numbers in the two columns indicate a sequence type exactly same in one or several species, and correspond to the numbers in the phylogenetic tree presented in Figure 1.

AK106715

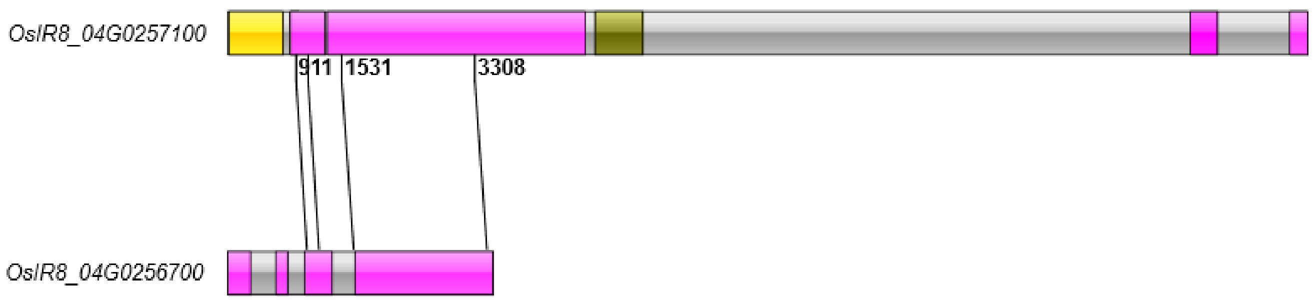

AK072107

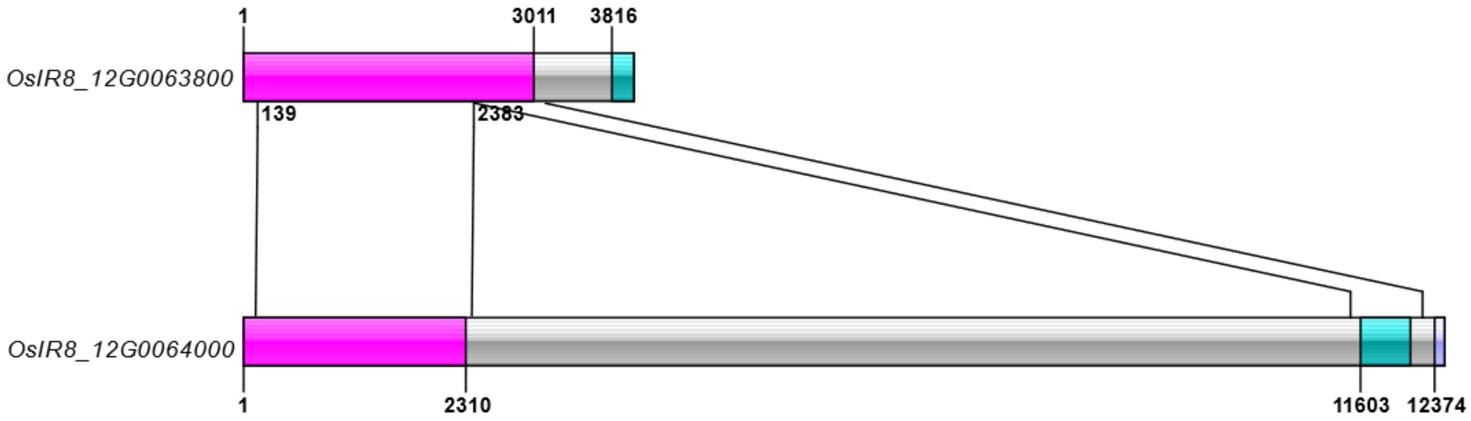

AK105722

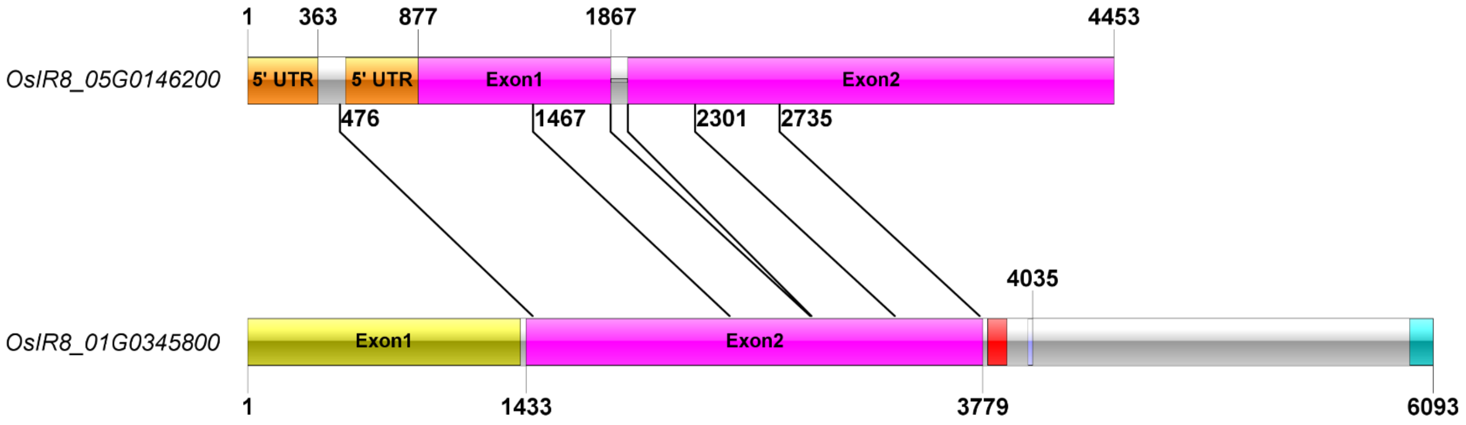

Figure S1. Chimeric retrogenes and the parental genes in *Indica* rice (IR8) genome. The sequences of the chimeric retrogenes and the corresponding parental genes were used as queries for blast searches of the IR8 genome, which was sequenced via PacBio technology.

AK070283

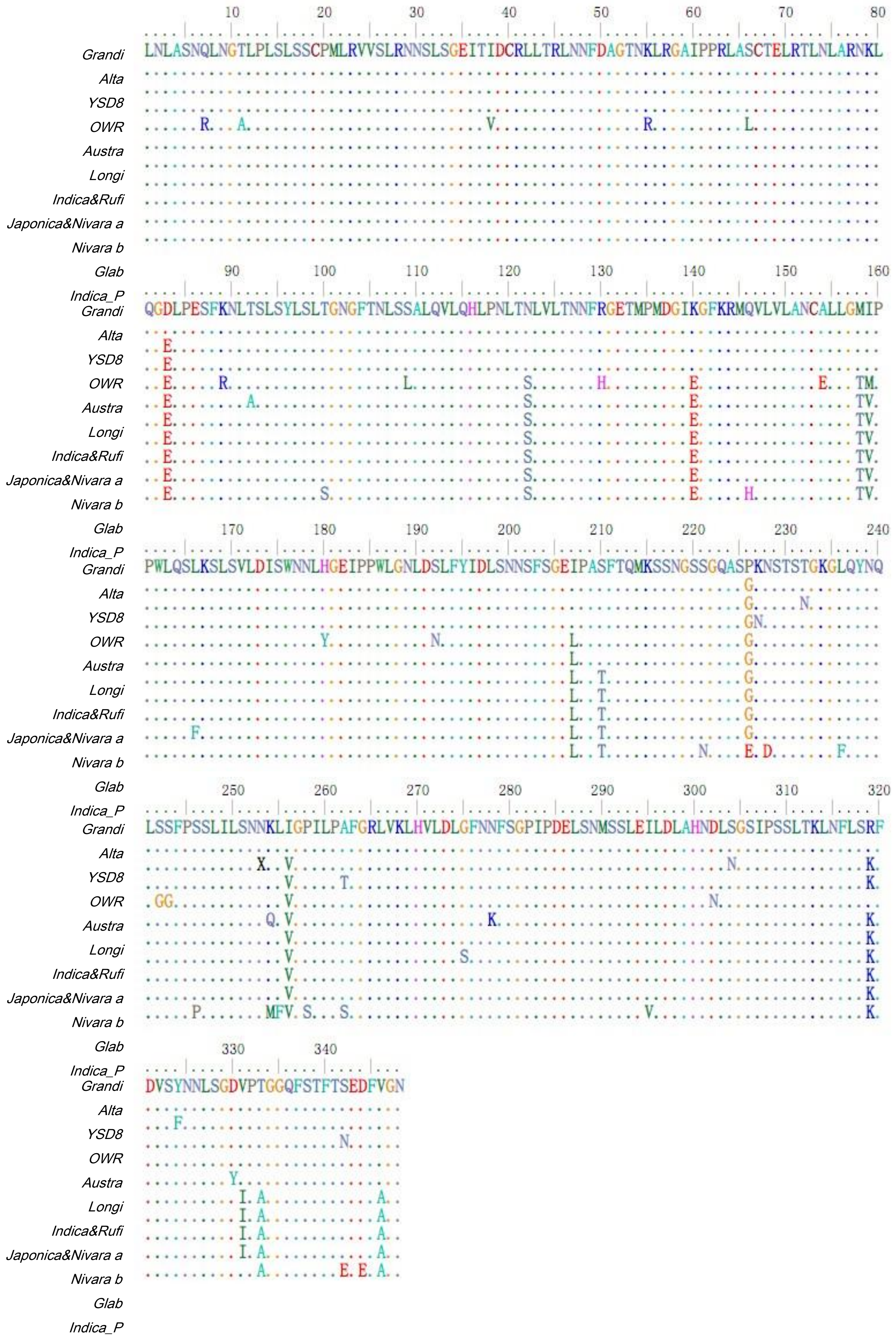
Grandi
Alta
YSD8
OWR
Austra
Longi
Indica&Rufi
Japonica&Nivara a
Nivara b
Glab
Indica_P
LNLASNQLNGTLPLSLSSCPMLRVVSLRNNSLSGEITIDCRLLTRLNNFDAGTNKLRGAIPPRLASCTELRTLNLARNKL
QGDLPESFKNLTSLSYLSLTGNGFTNLSSALQVLQHLPNLTNLVLTNNFRGETMPMDGIKGFKRMQVLVLANCALLGMIP
PWLQSLKSLSVLDISWNNLHGEIPPWLGNLDSLFYIDLSNNSFSGEIPASFTQMKSSNGSSGQASPKNSTSTGKGLQYNQ
LSSFPSSLILSNNKLIGPILPAFGRLVKLHVLDLGFNNFSGPIPDELSNMSSLEILDLAHNDLSGSIPSSLTKLNFLSRF
DVSYNNLSGDVPTGGQFSTFTSEDFVGN

`

AK073060

Japonica_P
Indica_P
Nivara b_P
Glab_P
Nivara a_P
Grandi_P
Alta_P
Nivara b&Indica&Longi&YSD8

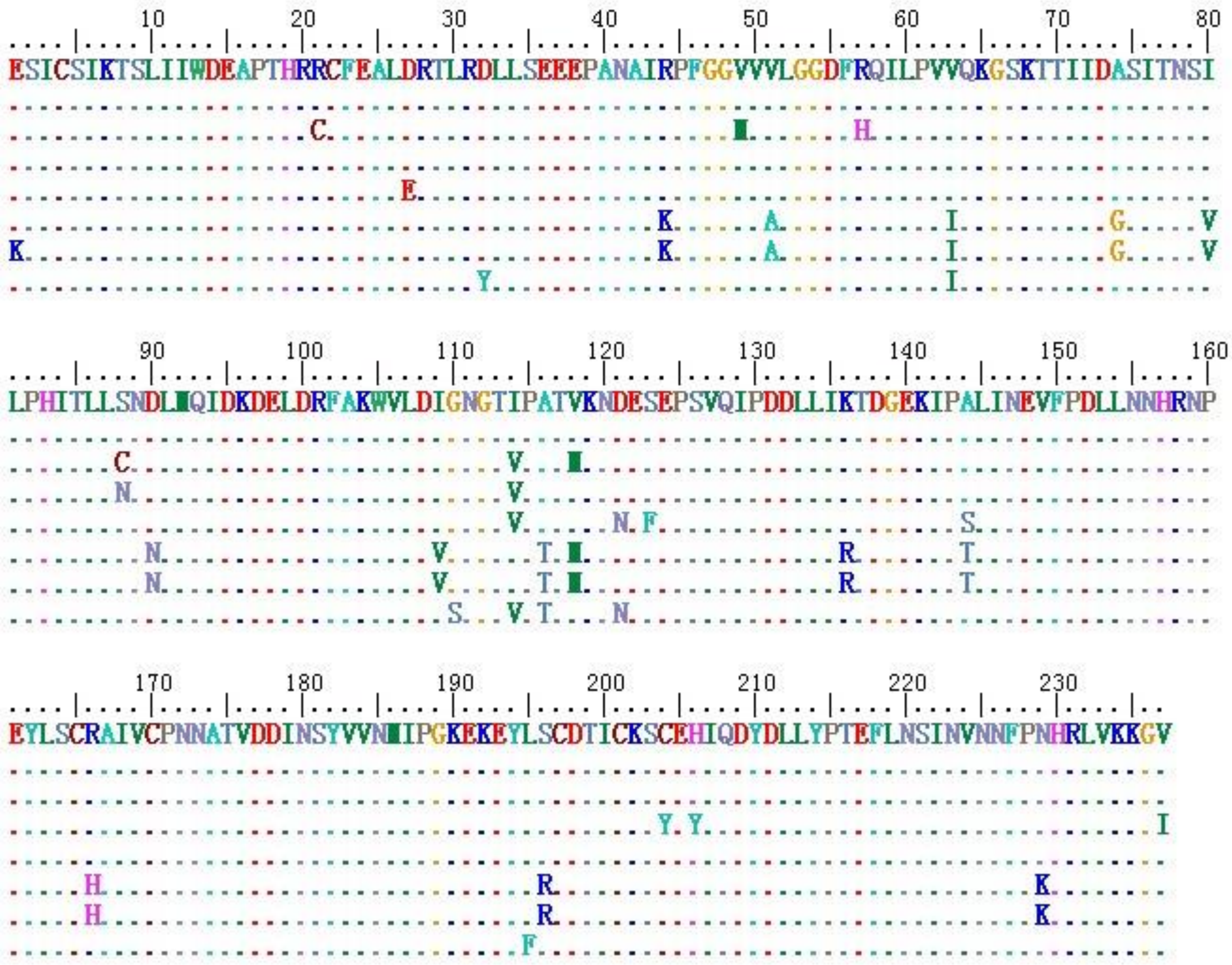

AK069420

Japonica
Indica
Longi
Nivara a&Nivara b
Rufi
Glab
Indica_P
Japonica_P

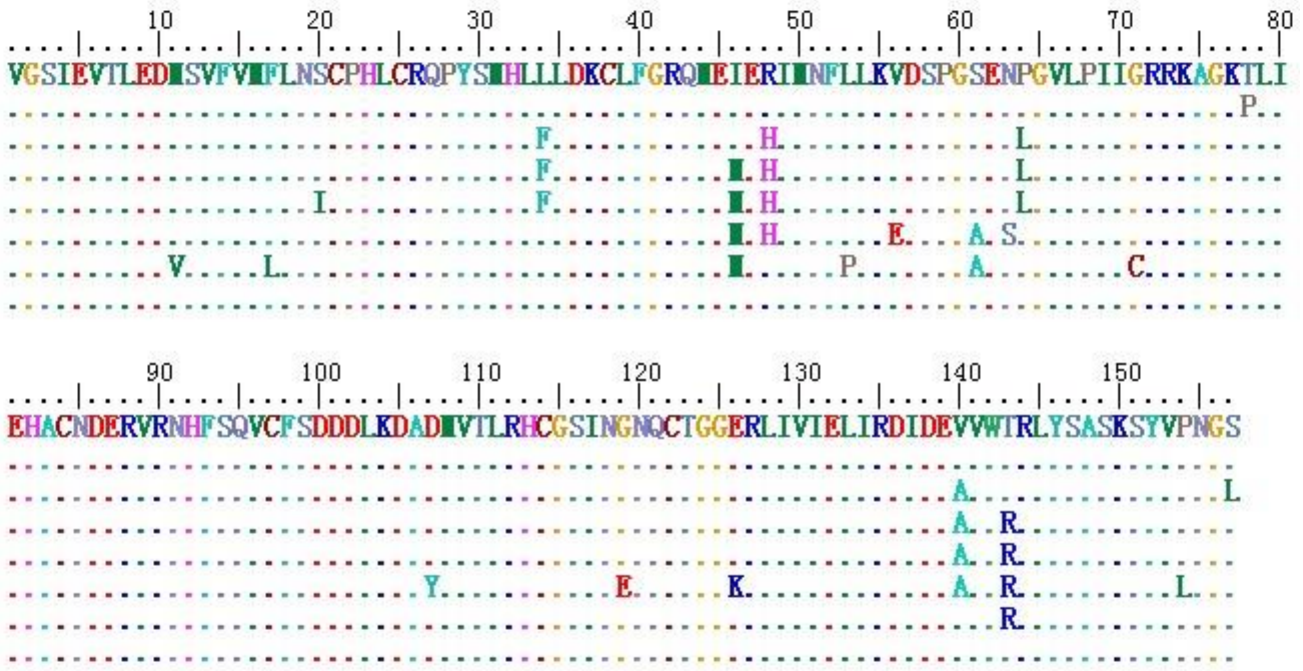

`

AK064415

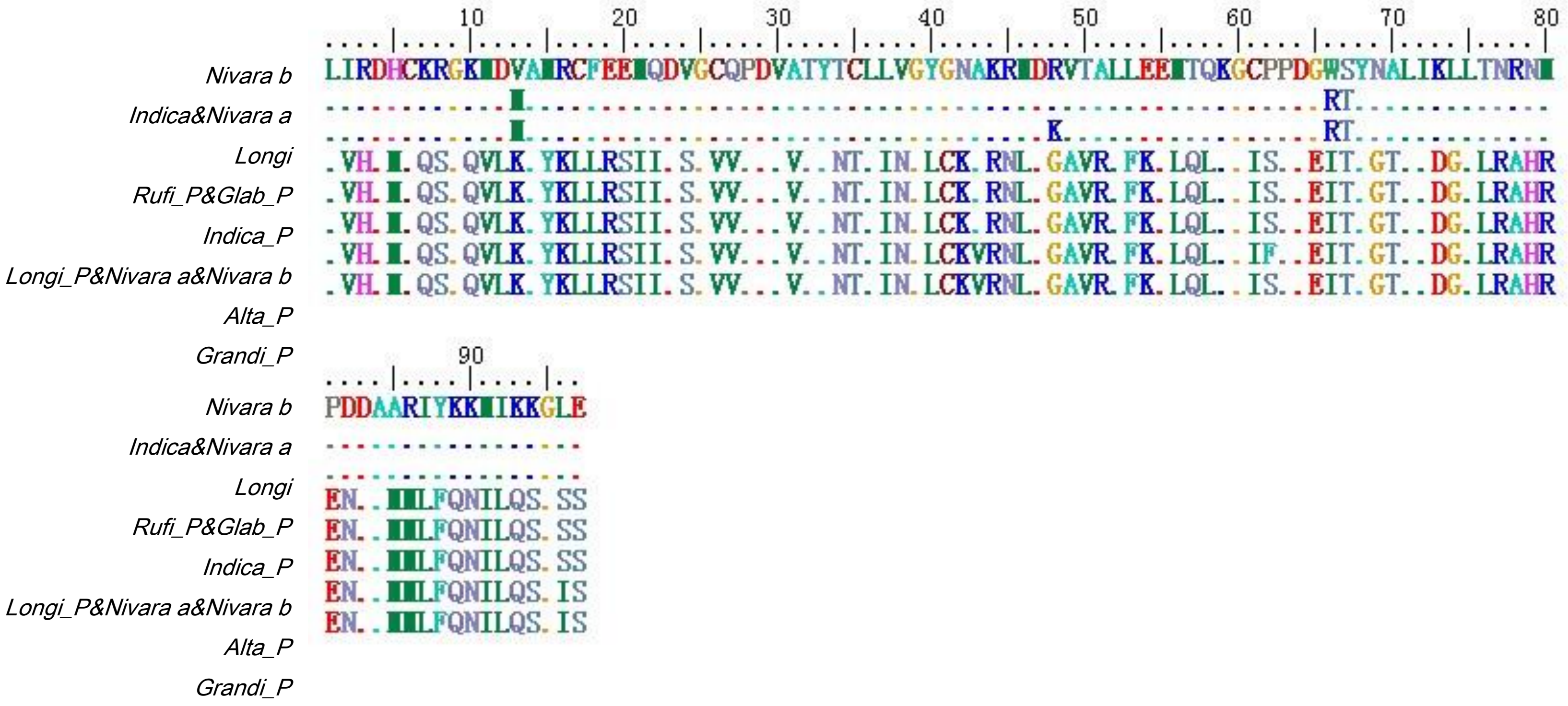

AK070367

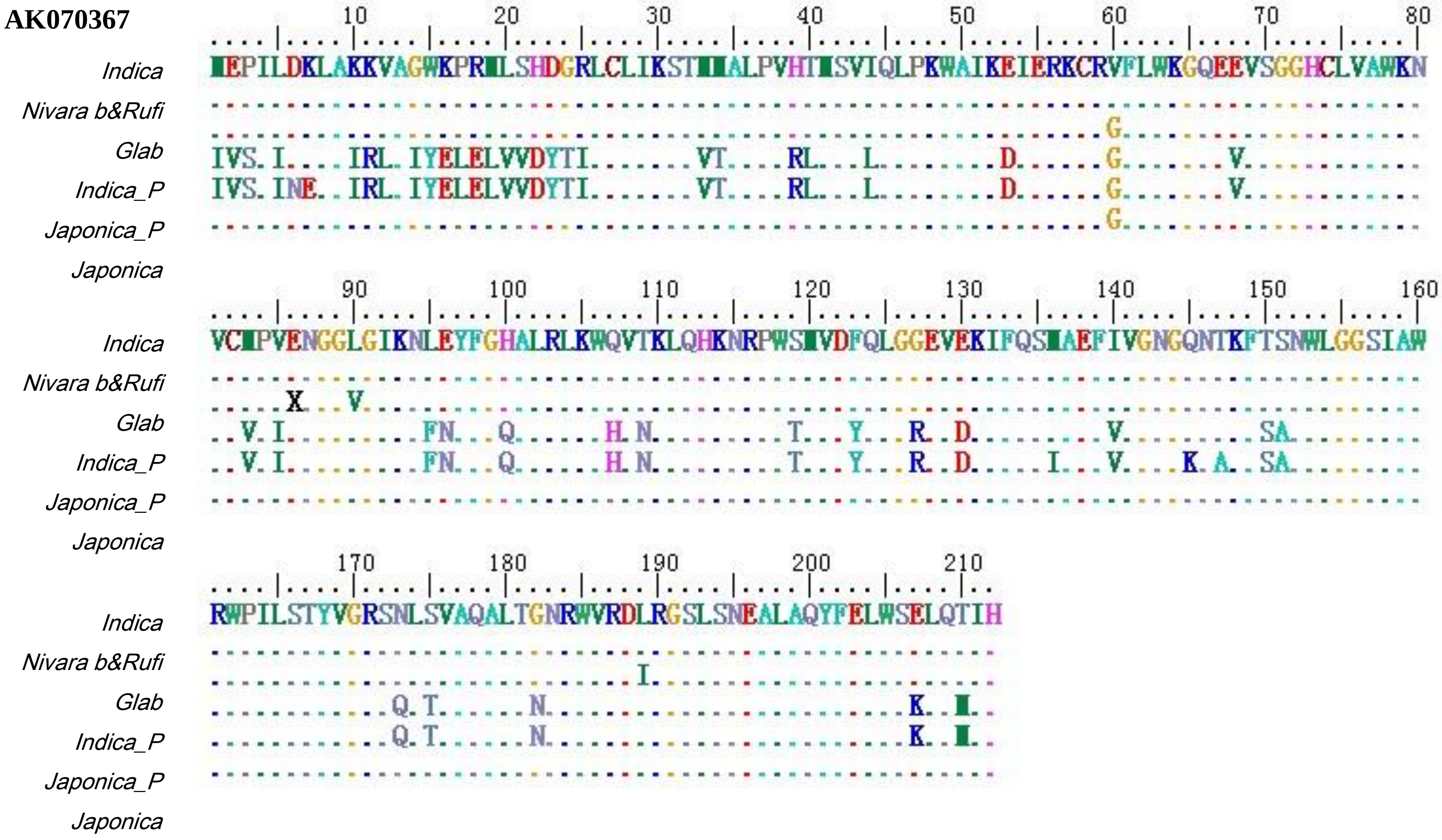

AK067652

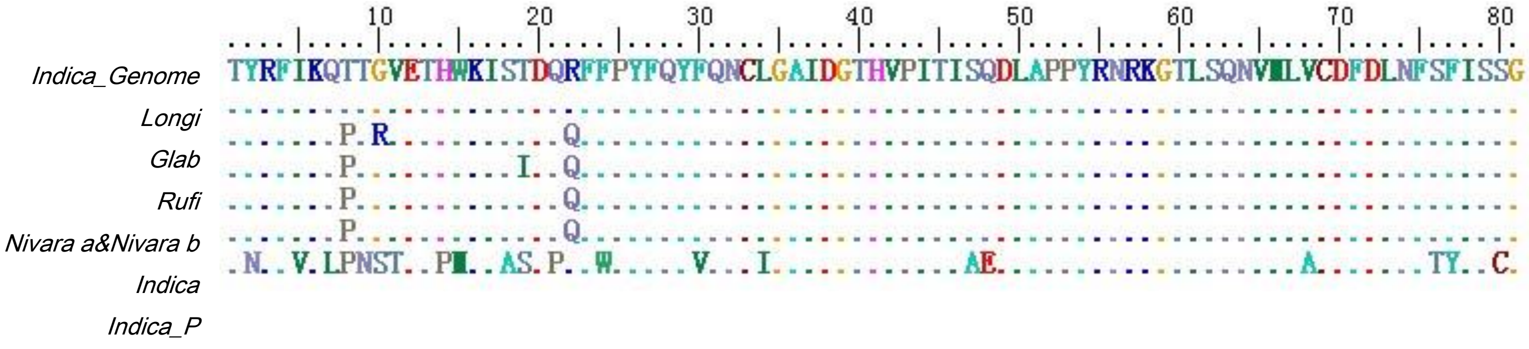

AK068903

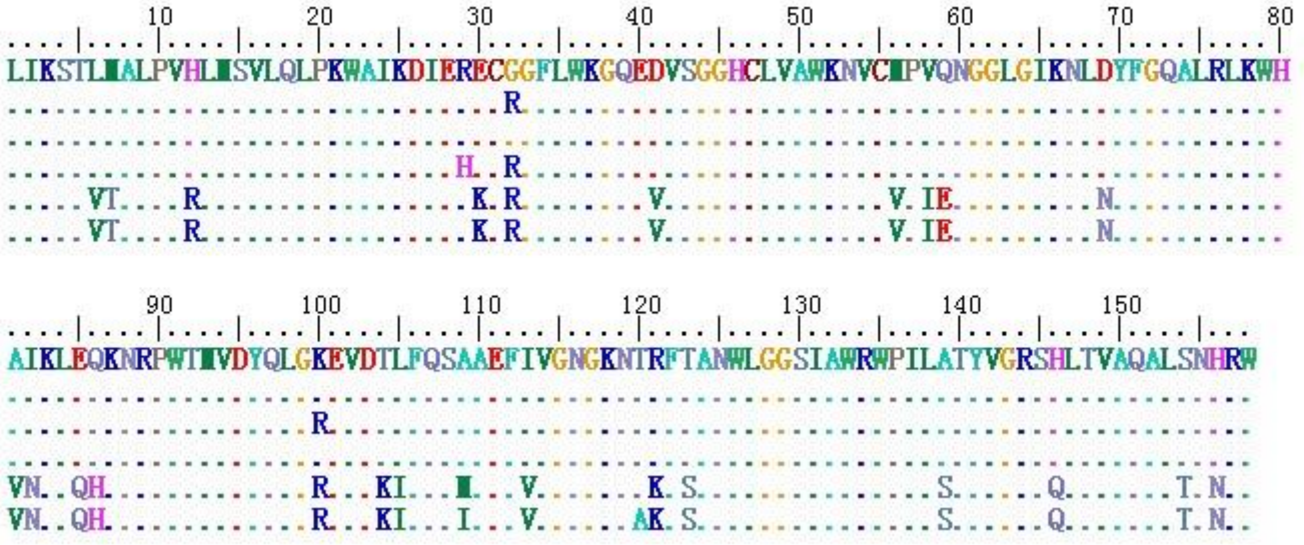

Rufi
Nivara a&Nivara b
Indica
Japonica
Indica_P
Japonica_P

AK070946

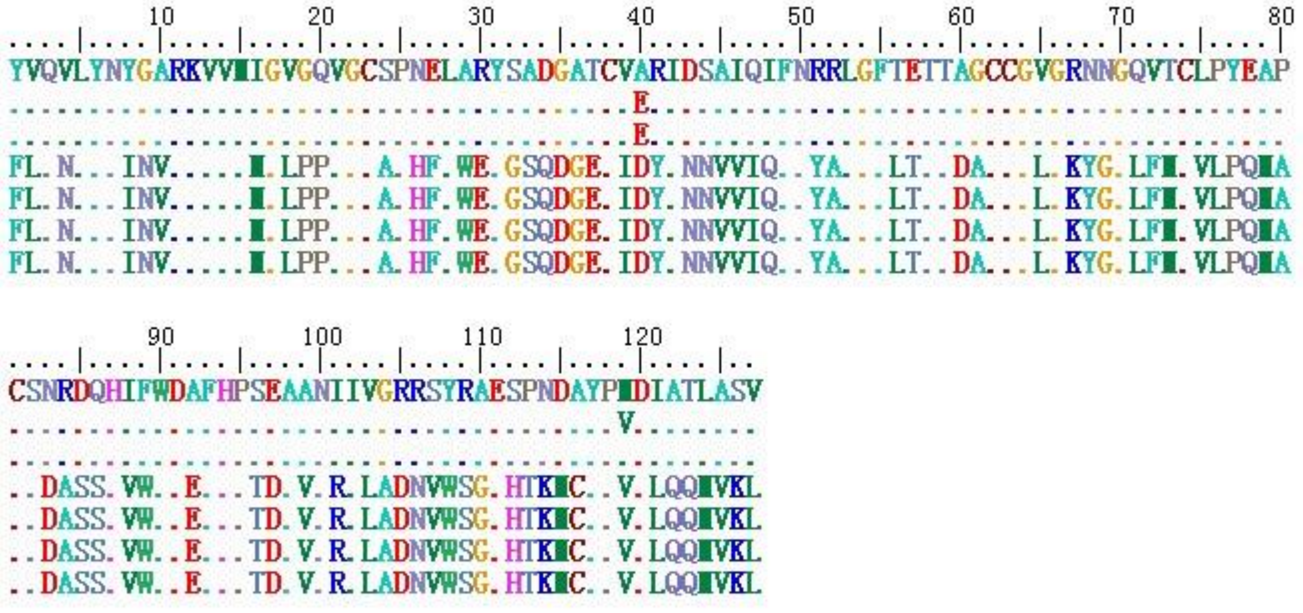

Indica&Glab&Rufi&Nivara a&Nivara b
Grandi&Alta
Austra
Indica_P&Nivra a_P&Nivara b_P
Rufi_P
Longi_P
Glab_P

AK073961

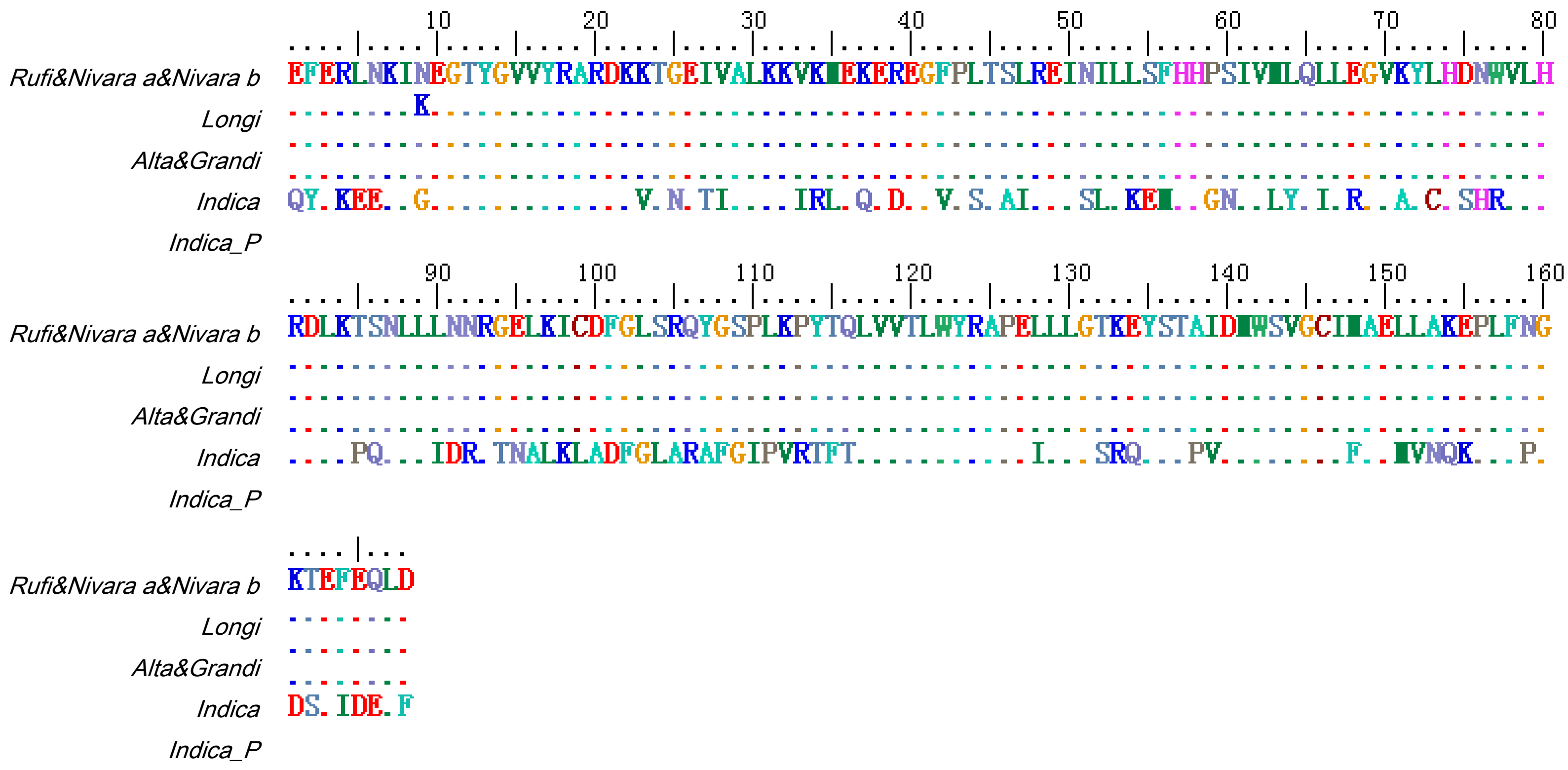

AK105360

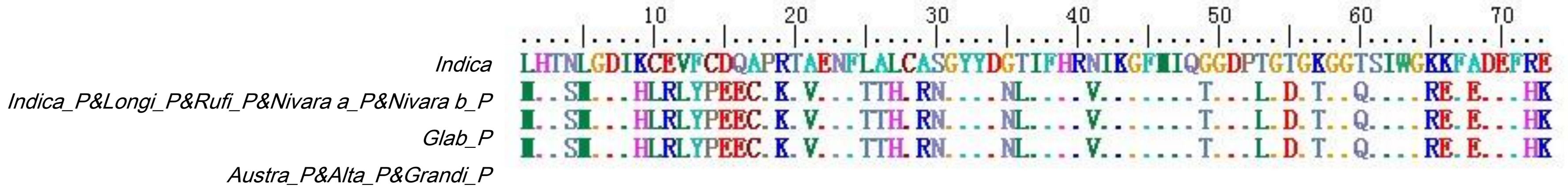

AK106308

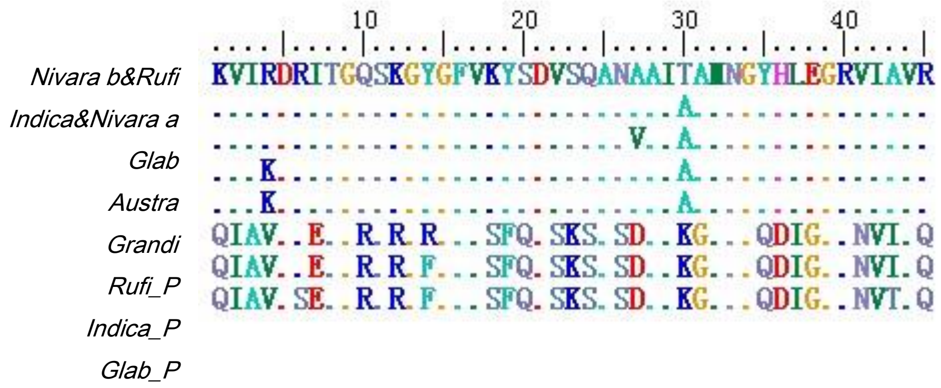

AK106445

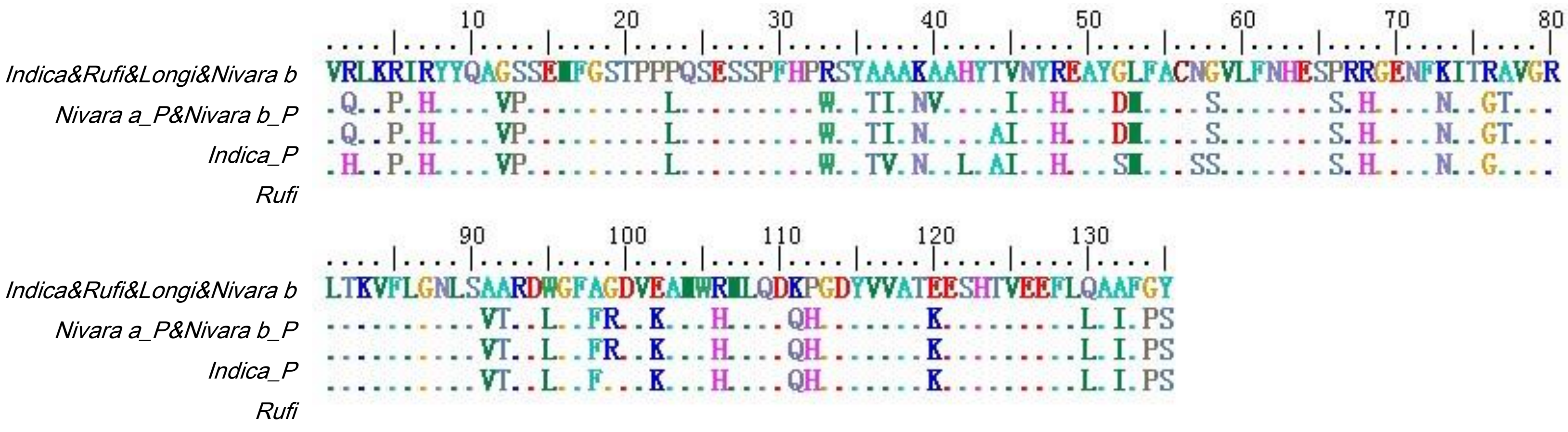

AK108373

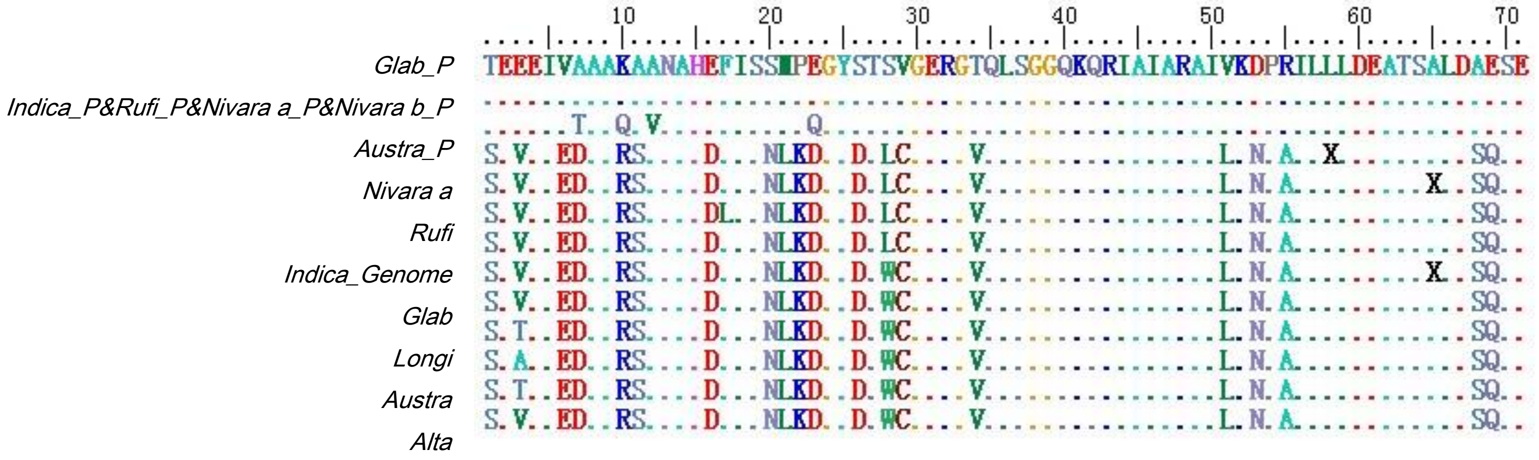

AK109583

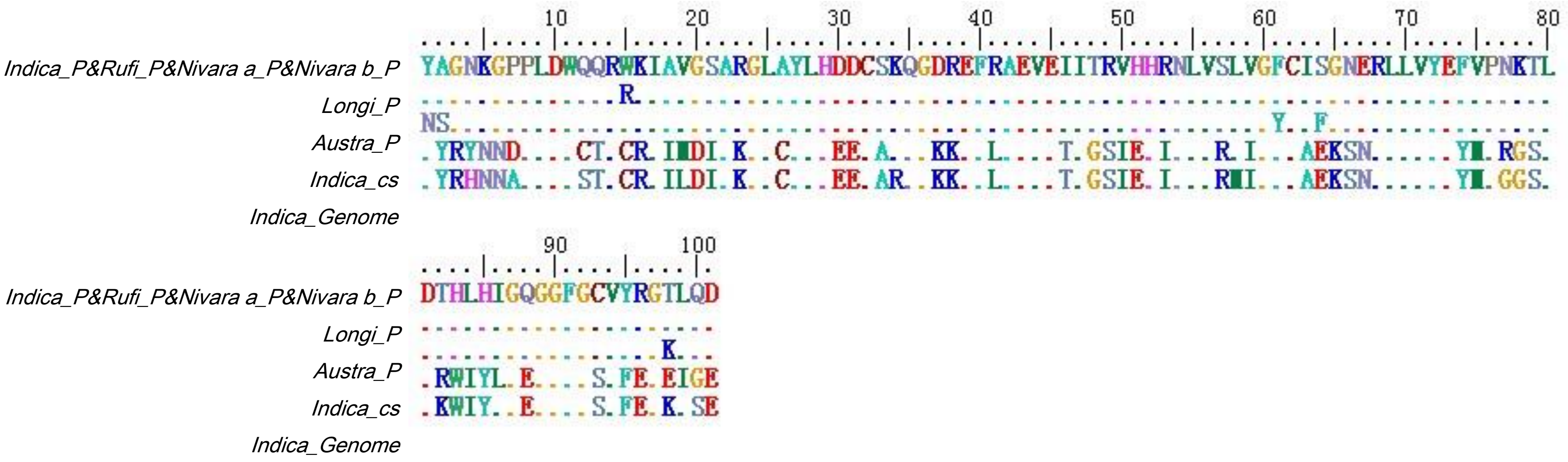

AK064216

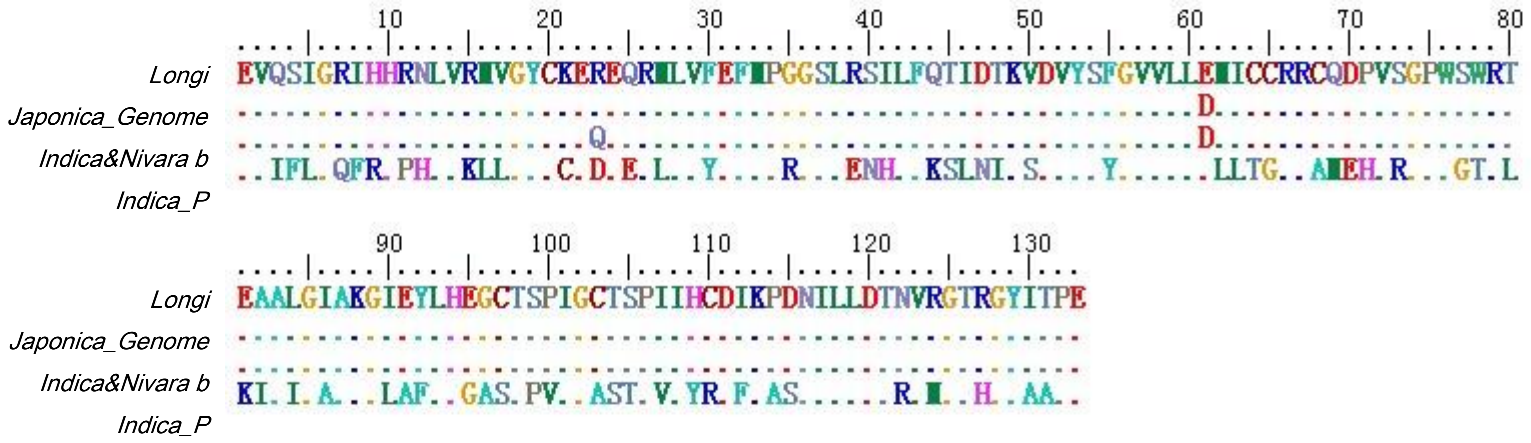

AK069257

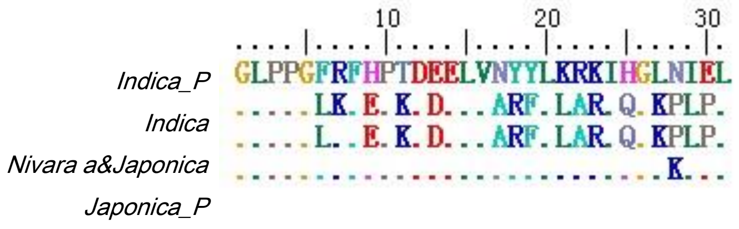

AK069587

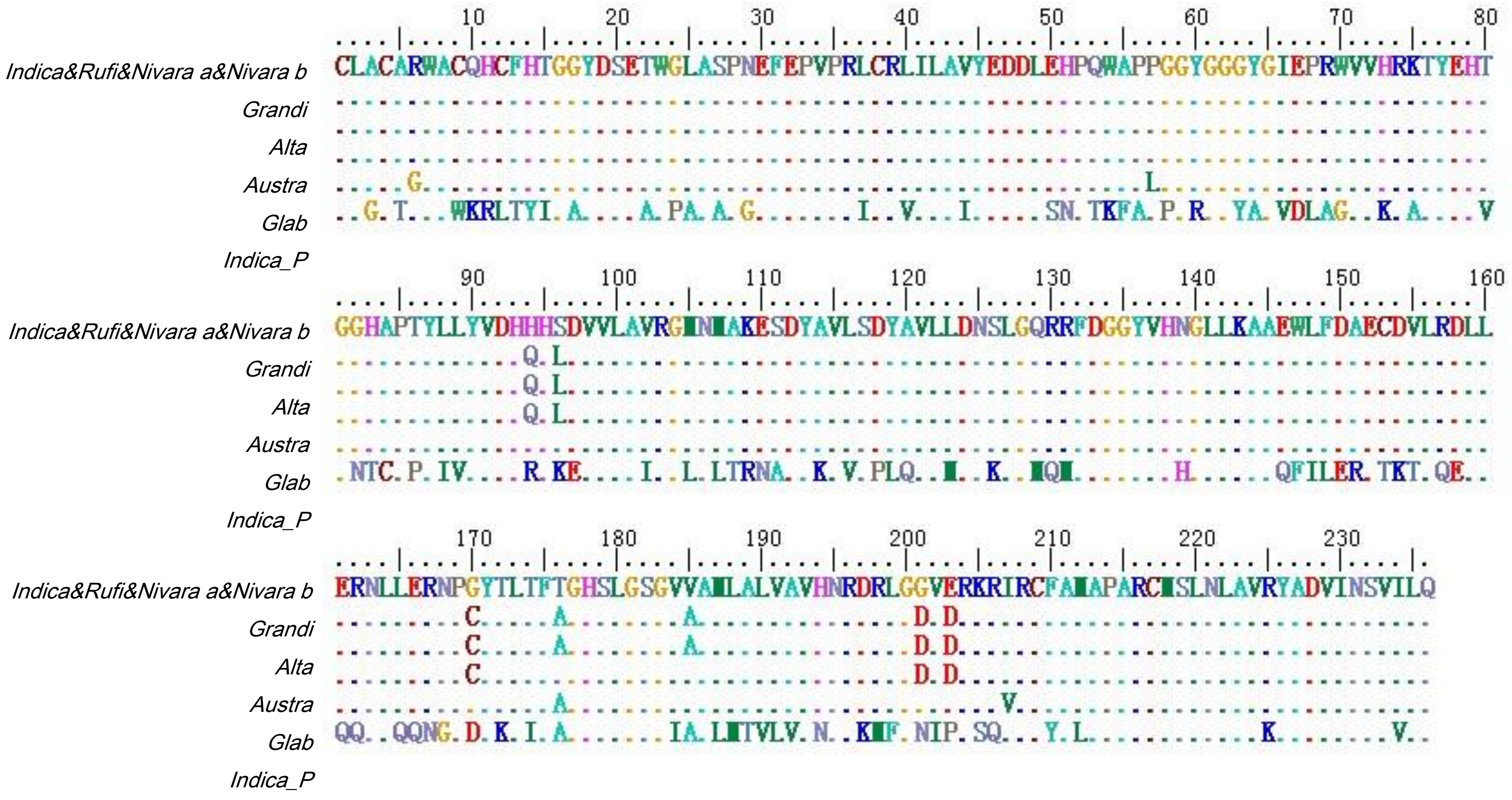

Figure S2. Alignment of the amino acid sequences of another 17 chimeric retrogene pairs

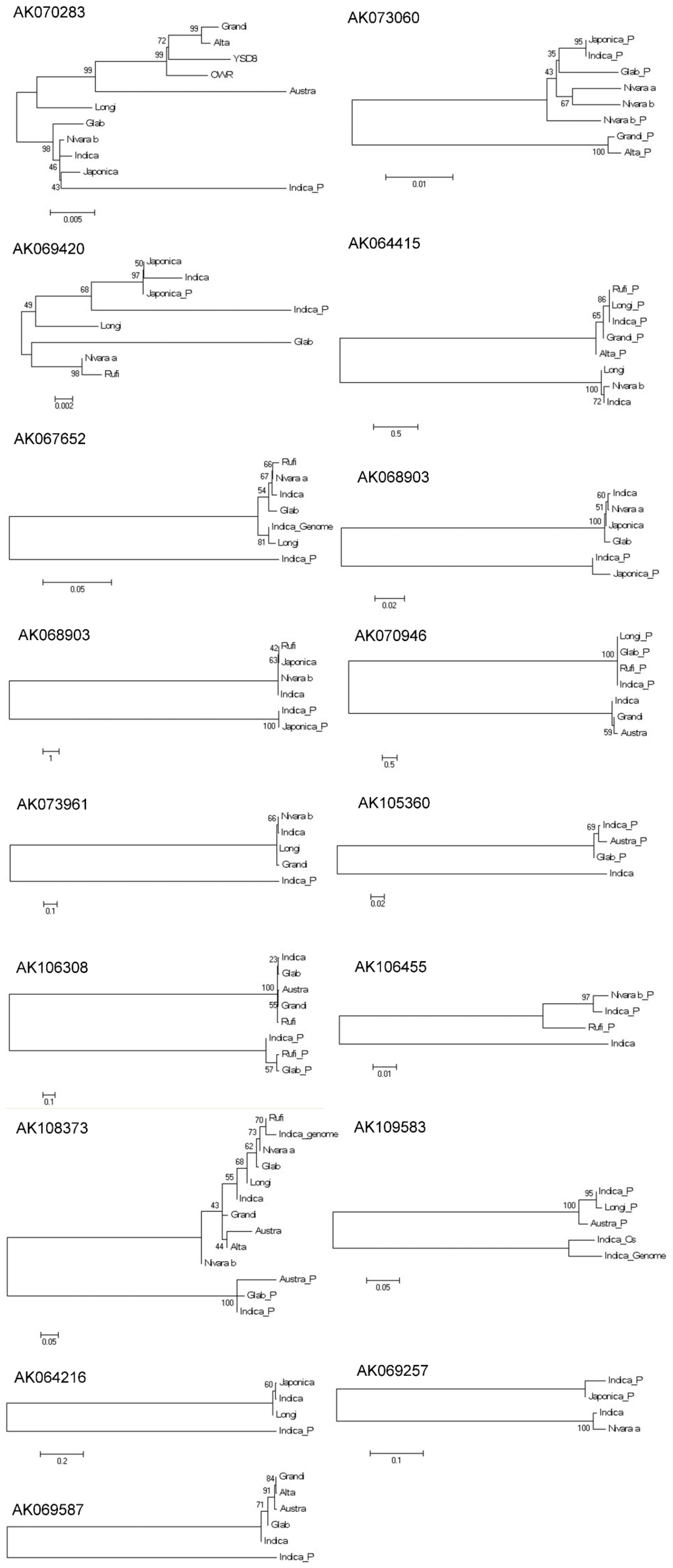

Figure S3. Phylogenetic relationships among 17 chimeric retrogene pairs. Refer to the Figure 1 legend for more details.

`
